# Exclusive Breastfeeding Drives AMPK‐Dependent Thermogenic Memory in BAT and Promotes Long‐Term Metabolic Benefits in Offspring

**DOI:** 10.1002/advs.202508956

**Published:** 2025-12-19

**Authors:** Ningxi Wu, Anwen Yin, Xiang Yu, Mingxin Wang, Jiahui Zhang, Kerong Liu, Yutong Hou, Minkai Cao, Yiting Zhang, Xiaoxiao Zhang, Yao Gao, Xirong Guo, Le Zhang, Yun Li

**Affiliations:** ^1^ Department of Pediatric Laboratory Affiliated Children's Hospital of Jiangnan University Wuxi Children's Hospital Wuxi Key Laboratory of Genetic and Metabolic Diseases in Children Wuxi Jiangsu 214023 China; ^2^ Department of Neonatology Affiliated Children's Hospital of Jiangnan University Wuxi Children's Hospital Wuxi Jiangsu 214023 China; ^3^ Department of Cardiology The Affiliated Wuxi People's Hospital of Nanjing Medical University Wuxi People's Hospital Wuxi Medical Center Nanjing Medical University Wuxi Jiangsu 214023 China; ^4^ Department of Biochemistry and Molecular Cell Biology Shanghai Key Laboratory for Tumor Microenvironment and Inflammation Shanghai Jiao Tong University School of Medicine Shanghai 200025 China; ^5^ Department of Endocrinology Affiliated Children's Hospital of Jiangnan University Wuxi Children's Hospital Wuxi Jiangsu 214023 China; ^6^ Department of Obstetrics and Gynecology Affiliated Women’s Hospital of Jiangnan University, Wuxi Maternity and Child Health Care Hospital Wuxi Jiangsu 214002 China; ^7^ Department of Endocrinology Children's Hospital of Nanjing Medical University Nanjing Jiangsu 210008 China; ^8^ Hongqiao International Institute of Medicine Tongren Hospital Shanghai Jiao Tong University School of Medicine Shanghai 200336 China

**Keywords:** AMPK, breastfeeding, brown adipose tissue, childhood obesity, α‐ketoglutarate

## Abstract

Exclusive breastfeeding reduces the risk of childhood obesity, potentially through metabolic programming of adipose tissue during lactation. However, the underlying mechanisms remain unclear. Using a mouse model, it is shown that mixed formula feeding disrupts brown adipose tissue (BAT) morphology, mitochondrial integrity, and thermogenic capacity, resulting in greater fat accumulation and glucose intolerance after weaning under a high‐fat diet. By contrast, BAT from exclusively breastfed mice preserved enhanced thermogenic function for up to 12 weeks after transplantation into recipient mice. Transcriptomic analysis revealed that AMPK activation is sustained in BAT from exclusively breastfed mice but markedly diminished in mixed‐fed counterparts. Pharmacological inhibition of AMPK abolished the long‐term metabolic benefits conferred by exclusive breastfeeding. Mechanistically, breast milk–derived extracellular vesicles enriched in miR‐125a‐5p enhanced AMPK signaling by targeting HIF1AN. AMPK‐induced α‐ketoglutarate (αKG) production proved essential for BAT development and thermogenesis, and αKG supplementation rescued impaired BAT function in mixed‐fed mice. In conclusion, exclusive breastfeeding imprints a thermogenic memory in BAT via the HIF1AN/AMPK/αKG signaling axis, thereby conferring long‐term metabolic protection to offspring.

## Graphical abstract

Exclusive breastfeeding establishes a thermogenic memory in brown adipose tissue by activating the HIF1AN/AMPK/α‐ketoglutarate axis via milk‐derived extracellular vesicles enriched in miR‐125a‐5p. This programming preserves metabolic health, while αKG supplementation restores BAT function under mixed feeding, offering strategies to mitigate the consequences of non‐exclusive breastfeeding.

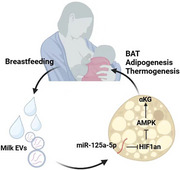

## 1 Introduction

The rates of childhood obesity have continued to increase, becoming one of the most serious global public health challenges.^[^ ^1^, ^2^ ^]^ Childhood obesity is associated with long‐term risks, including obesity in adulthood, chronic diseases, and increased mortality.^[^ ^3^ ^]^ Current advancements in understanding indicate that obesity is a multifactorial pathology influenced by environmental, genetic, and epigenetic factors.^[^ ^4^ ^]^ The rapid increase in the prevalence of childhood obesity may particularly result from early‐life determinants, such as maternal diet and neonatal feeding.^[^ ^5^, ^6^ ^]^

The pattern of infant feeding during the first 1000 days, including pregnancy and the first two years of postnatal life, plays a crucial role in a child's growth trajectory.^[^ ^7^, ^8^ ^]^ The association between exclusive breastfeeding and a reduced risk of childhood obesity has attracted considerable scientific attention, particularly as this form of malnutrition becomes a global epidemic. The World Health Organization (WHO) recommends exclusive breastfeeding for the first six months, followed by continued breastfeeding with appropriate complementary foods for up to two years or beyond.^[^ ^9^ ^]^ Cohort studies, including several meta‐analyses, have suggested that exclusive breastfeeding positively influences the risk of overweight and obesity, not only in childhood but also in adolescence and adulthood.^[^ ^10^, ^11^, ^12^, ^13^, ^14^, ^15^ ^]^ Bergman et al.^[^ ^16^ ^]^ conducted a longitudinal birth cohort study to investigate whether exclusive breastfeeding was correlated with BMI at six years of age. By three months, bottle‐fed infants had significantly higher BMIs and thicker skinfolds compared to breastfed infants. Between ages 4 and 6, the prevalence of obesity in bottle‐fed children nearly doubled and then tripled. Early bottle feeding appears to accelerate the obesity rebound, a key predictor of obesity later in life. However, in a large cohort of children followed prospectively from birth, Huus et al.^[^ ^17^ ^]^ found that while short‐term exclusive breastfeeding showed a trend toward an association with obesity at five years of age (P = 0.05), this association was no longer statistically significant when potential confounders, such as socioeconomic factors, were accounted for. Currently, the causal relationship between exclusive breastfeeding and reduced childhood obesity risk requires stronger evidence for confirmation.

Studies suggest that both breastfeeding patterns and the bioactive components in breast milk contribute to the metabolic benefits of breastfeeding in offspring. Breastfeeding duration is a critical parameter influencing long‐term metabolic outcomes. Early weaning, induced by interrupting maternal lactation, resulted in weanling rat offspring with lower body weight. However, after catch‐up growth, the adult offspring developed hypothalamic leptin resistance, along with hyperphagia, increased body weight, and adiposity.^[^ ^18^ ^]^ In contrast, Pena‐Leon et al.^[^ ^6^ ^]^ found that delayed weaning in rat pups protects against diet‐induced obesity in adulthood by enhancing brown adipose tissue thermogenesis and energy expenditure, mediated through liver‐to‐hypothalamus communication. Additionally, breast milk is rich in various bioactive factors, including microRNAs (miRNAs), lipokines/signaling lipids, small molecules/metabolites, and fructose, which may modulate mechanisms involved in controlling energy homeostasis.^[^ ^19^ ^]^ The nutritional alterations, such as a high‐fat diet in the dam during the lactation–suckling period, change the composition of maternal milk and affect the obesity programming in the offspring.^[^ ^20^, ^21^ ^]^ Rat offspring raised artificially via gastrostomy and fed an isocaloric high‐carbohydrate milk formula (as opposed to fat‐rich rat milk) exhibited persistent hyperphagia and increased adiposity.^[^ ^22^ ^]^ Yu et al. suggested that the lipid group of alkylglycerols that is specific to breast milk promotes infant beige adipose tissue development.^[^ ^23^ ^]^ Despite these insights, the mechanisms underlying the long‐lasting metabolic benefits of breastfeeding remain elusive.

Metabolic programming refers to the process by which nutritional stress or stimuli during critical periods of early development permanently alter an organism's physiology and metabolism, with the consequences often manifesting much later in life.^[^ ^24^ ^]^ However, the specific imprinting mechanisms by which breast milk exerts protective effects over formula remain largely unexplored. In this study, we established a mouse model comparing exclusive breastfeeding (EB) and mixed feeding (MF) groups. MF mice exhibited abnormal brown adipose tissue (BAT) development and heightened sensitivity to high‐fat diet (HFD)‐induced metabolic dysfunction after weaning. We found that miR‐125a‐5p in breast milk extracellular vesicles (EVs) activates the AMPK/αKG pathway by inhibiting HIF1AN, thereby increasing PRDM16 expression to promote brown adipogenesis and UCP1 expression to enhance thermogenesis. Notably, EB mice exhibited prolonged AMPK activity in BAT compared to MF mice after weaning, suggesting a form of metabolic memory that may contribute to sustained metabolic health in offspring.

## 2 Results

### 2.1 Exclusive Breastfeeding Contributes to BAT Development

**Figure 1:**
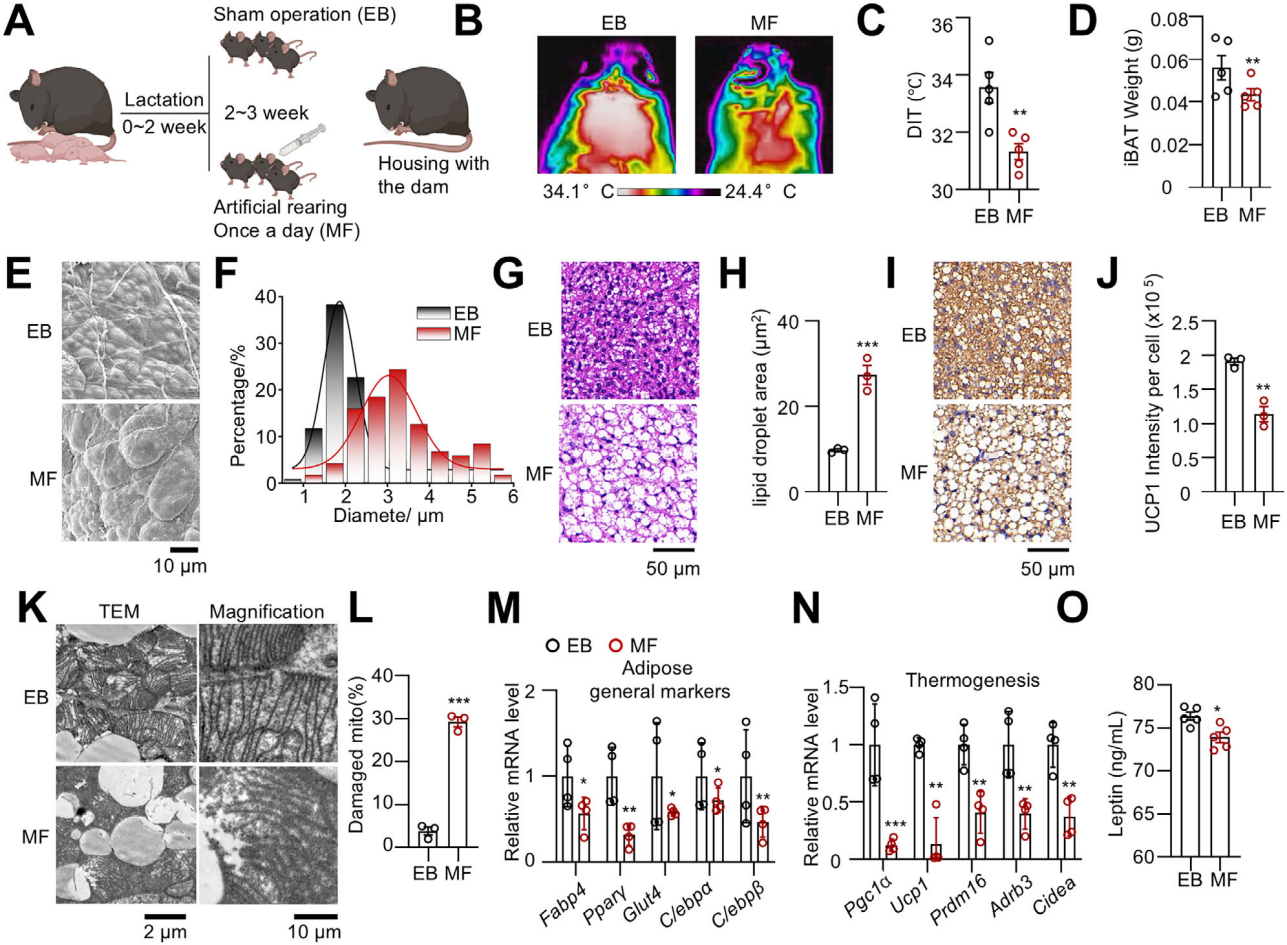
Exclusive breastfeeding contributes to BAT development. A) Schematic representation of the exclusive breastfeeding (EB) and mixed‐feeding (MF) mouse models. Newborn male mice were breastfed from birth to 2 weeks of age. At 2 weeks, MF mice received artificial formula once daily before being returned to their mothers for breastfeeding. EB mice were exclusively breastfed but underwent the same sham feeding procedure once daily. B, C) Thermal imaging of the intrascapular region in EB and MF mice following 1 h of cold exposure at 4 °C. (n = 5). D) The iBAT weight in EB and MF mice. (n = 5). E, F) Scanning electron microscopy images of iBAT at 3 weeks (E) and lipid droplet size distribution (F). G–J) H&E staining (G, H) and UCP1 IHC analysis (I, J) of iBAT. (n = 3). K, L) TEM images of iBAT, showing mitochondrial morphology. (n = 3). M, N) qRT‐PCR analysis of adipose tissue markers and thermogenesis‐related gene expression in iBAT of EB and MF mice. (n = 4). O) The serum leptin level was measured in EB and MF mice. (n = 5). Data are presented as mean ± SEM. Statistical significance was determined using a two‐tailed Student's *t*‐test (^*^ *p* < 0.05; ^**^ *p* < 0.01; ^***^ *p* < 0.001).

To determine the role of exclusive breastfeeding in metabolic regulation of offspring, we partially replaced maternal milk with formula during the last week of lactation (postnatal days 14–21) by artificially feeding mouse pups using specially designed nipples, which minimized stress (**Figure** **1**A; Figure S1A, Supporting Information). During this artificial feeding period, no significant difference in body weight gain was observed between exclusively breastfed (EB) and mixture‐fed (MF) pups (Figure S1B, Supporting Information). However, upon cold exposure at 4 °C, EB pups maintained both whole‐body and dorsal interscapular temperatures (DIT) more effectively than MF pups (Figure 1B,C; Figure S1C, Supporting Information).

Dissection revealed that, although there were no significant differences in inguinal white adipose tissue (iWAT), epididymal white adipose tissue (eWAT), or lean body mass between groups, MF pups displayed reduced interscapular brown adipose tissue (iBAT) mass compared to their EB littermates (Figure 1D; Figure S1D–G, Supporting Information). Notably, thermogenic gene expression in iBAT, such as *Ucp1* and *Prdm16*, gradually increased during the lactation period but rapidly declined after weaning (Figure S1J,K, Supporting Information). Specifically, UCP1 levels gradually increased after birth, reached markedly elevated levels at weeks 2–3, and declined after weaning, consistent with previous findings^[^ ^25^ ^]^ (Figure S1H,I, Supporting Information).

To further elucidate the impact of exclusive breastfeeding on BAT biology, scanning electron microscopy was performed and revealed that EB mice exhibited smaller, more numerous, and more uniformly distributed lipid droplets in iBAT compared to MF mice (Figure 1E,F). Histological analysis with H&E staining confirmed these morphological differences (Figure 1G,H). Immunohistochemical staining further showed elevated UCP1 expression in the iBAT of EB pups relative to MF pups (Figure 1I,J). Transmission electron microscopy (TEM) revealed that mitochondrial density was lower and cristae were poorly organized in MF pups (Figure 1K,L). In line with these findings, the mRNA expression levels of key adipogenic and thermogenic markers were significantly reduced in MF pups compared to EB pups (Figure 1M,N).

Moreover, we observed higher serum leptin levels in EB mice (Figure 1O). However, food intake between MF and EB pups showed no significant differences (Figure S1L, Supporting Information).

### 2.2 Exclusive Breastfeeding Protects Offspring Against High‐Fat Diet‐Induced Adiposity

**Figure 2:**
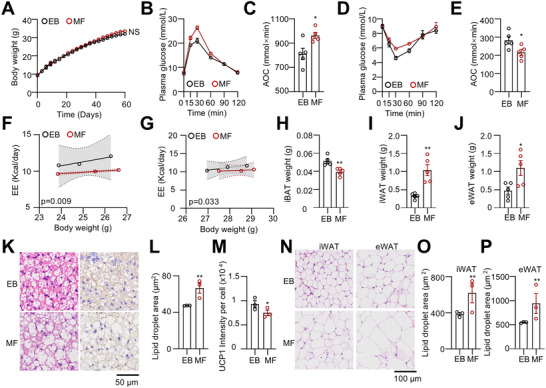
Exclusive breastfeeding protects offspring against high‐fat diet‐induced adiposity. A) Body weight of EB and MF male mice challenged with a 45% HFD (n = 5). B, C) Glucose tolerance test (GTT) and area of the curve (AOC) analysis in EB and MF mice following HFD. (n = 5). D, E) Insulin tolerance test (ITT) and AOC analysis in EB and MF mice after HFD. (n = 5). F, G) Covariate analysis (ANCOVA) of energy expenditure (EE) versus body weight in EB and MF mice following HFD challenge under room temperature (25 °C) (F) and thermoneutral conditions (30 °C) (G). (n = 3). H–J) The weight of iBAT, iWAT, and eWAT in EB and MF mice after HFD treatment was assessed. (n = 5). K–M) HE staining, UCP1 immunohistochemistry, and quantification of iBAT (n = 3). N–P) HE staining and lipid droplet size quantification in iWAT and eWAT (n = 3). Data are presented as mean ± SEM. Statistical significance was determined by two‐way ANOVA for (A), ANCOVA with body weight as a covariate for energy expenditure analyses (F, G), and two‐tailed Student's t‐test for all other panels (^*^ *p* < 0.05, ^**^ *p* < 0.01, ^***^ *p* < 0.001).

To investigate whether exclusive breastfeeding has a lasting impact on offspring metabolism, we examined the susceptibility of EB and MF pups to high‐fat diet (HFD)‐induced obesity. After weaning, EB and MF pups were maintained on a 45% HFD for two months. There were no significant differences in body weight gain or food intake between the groups (**Figure** **2**A; Figure S2A, Supporting Information). Notably, EB+HFD mice exhibited improved glucose tolerance and enhanced insulin sensitivity compared to MF+HFD mice (Figure 2B–E). Additionally, energy expenditure was lower in MF+HFD mice than in EB+HFD mice, despite no significant differences in locomotor activity (Figure 2F; Figure S2B, Supporting Information). In a cold‐exposure test, EB+HFD mice maintained DIT more effectively than MF+HFD mice at 4 °C (Figure S2C,D, Supporting Information). To minimize the contribution of sympathetically driven BAT thermogenesis to energy expenditure, we further evaluated both groups under thermoneutral conditions (30 °C). Notably, the difference was attenuated compared with that observed at room temperature (Figure 2G).

After sacrifice, we observed that MF+HFD mice had a lower iBAT mass but higher iWAT and eWAT masses compared to EB+HFD mice (Figure 2H–J). However, there was no significant difference in lean body weight between the two groups (Figure S2E, Supporting Information). Histological analysis revealed that EB+HFD mice had smaller lipid droplets and higher UCP1 content in iBAT compared to MF+HFD mice (Figure 2K–M). Similar findings were observed in iWAT and eWAT (Figure 2N–P).

We also examined the metabolic effects of exclusive breastfeeding in female mice. Consistent with male offspring, exclusive breastfeeding did not significantly alter HFD‐induced body weight gain in females compared with mixed formula feeding (Figure S2F, Supporting Information). However, EB females exhibited modest improvements, including enhanced energy expenditure, increased BAT mass, and elevated UCP1 content, although these effects were less pronounced than those observed in males (Figure S2G–K, Supporting Information). Moreover, GTT and ITT were not significantly improved in EB females compared with MF controls (Figure S2L–O, Supporting Information).

### 2.3 Exclusive Breastfeeding Drives Thermogenic Memory in Offspring BAT

**Figure 3:**
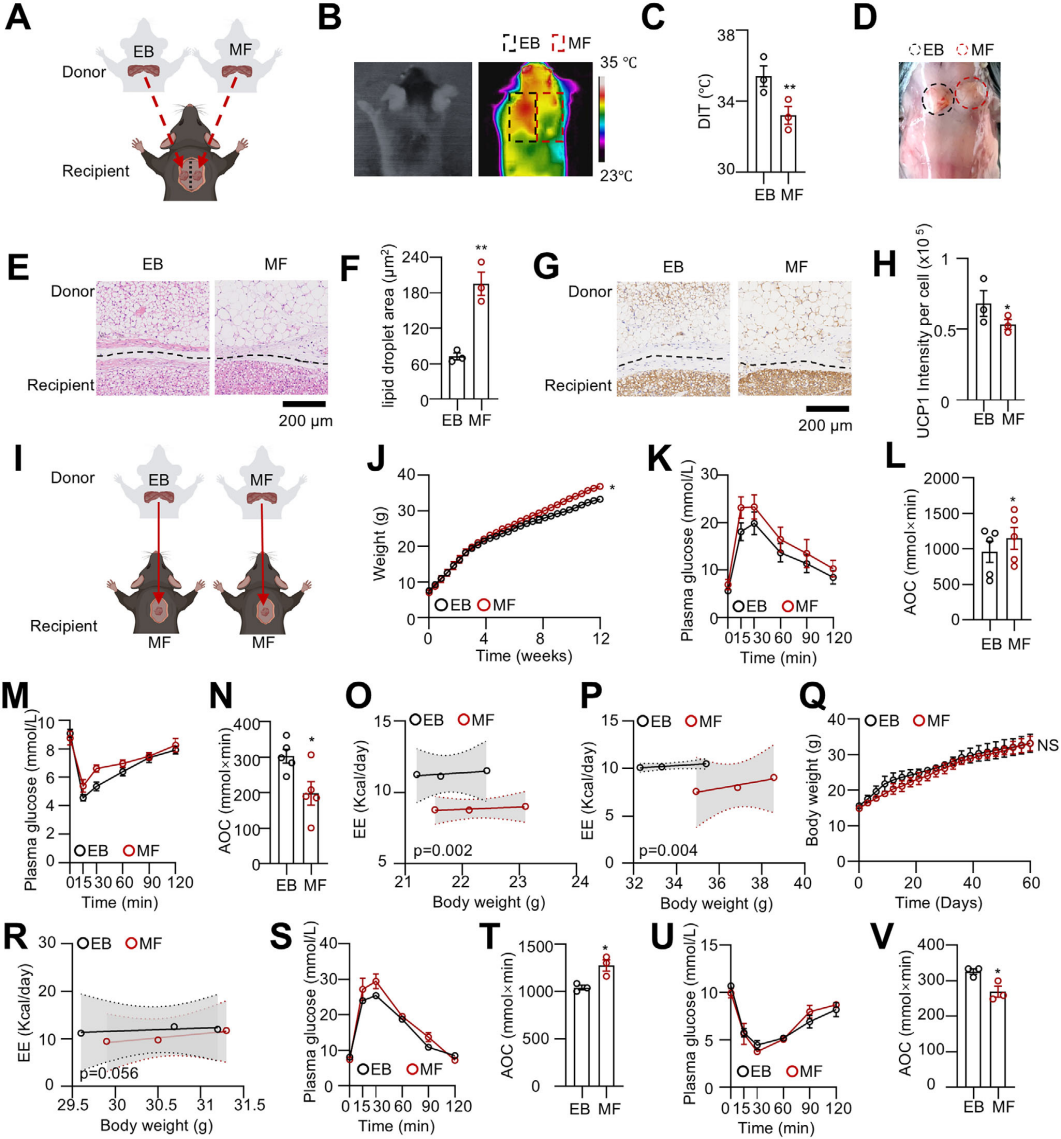
Exclusive breastfeeding imprints thermogenic programming in offspring BAT. A) Schematic diagram of iBAT transplantation in male mice: 10 mg of iBAT was harvested from 3‐week‐old EB and MF mice and transplanted into each side of the scapular region of the same 3‐week‐old EB mice. B, C) Twelve weeks after transplantation, the DIT was measured under 4 °C cold stimulation. (n = 3). D) Photos of the interscapular region showing successful transplantation of bilateral iBAT. E, F) HE staining was used to assess the histological characteristics of iBAT in recipient mice. (n = 3). G, H) UCP1 immunohistochemistry was performed to evaluate UCP1 levels in iBAT of recipient mice. (n = 3). I) Schematic diagram of iBAT transplantation in male mice: 10 mg of iBAT was taken from 3‐week‐old EB and MF mice and separately transplanted into the interscapular region of 3‐week‐old MF mice. J) Transplanted mice were fed a 45% HFD, and body weight was monitored. (n = 5). K–N) After 12 weeks of HFD, GTT (K,L) and ITT (M‐N) were determined. (n = 5). O–P) At week 4 (O) and week 12 (P), EE were measured. (n = 3). Q) Body weight of iBAT‐removed male mice fed a 45% HFD (n = 3). R) Energy expenditure in iBAT‐ablated EB and MF mice (n = 3). S–V) GTT (S, T) and ITT (U, V) performed in iBAT‐ablated mice from EB and MF groups after 2 months of HFD feeding (n = 3). Data are presented as mean ± SEM. Statistical significance was determined by two‐way ANOVA for (J, Q), ANCOVA with body weight as a covariate for energy expenditure analyses (O, P, R), and two‐tailed Student's *t*‐test for all other panels (^*^ *p* < 0.05, ^**^ *p* < 0.01, ^***^ *p* < 0.001).

A key question arises: why do EB mice continue to exhibit greater resistance to HFD‐induced obesity even eight weeks after weaning compared to MF mice? We hypothesized that this may be due to the direct programming of BAT by exclusive breastfeeding. To test this, we conducted BAT transplantation following established protocols.^[^ ^26^ ^]^ As illustrated in **Figure** **3**A, equal masses of iBAT from EB and MF donor mice were transplanted into the intrascapular region of recipient mice (EB mice), which were then maintained on a chow diet. Remarkably, in the same recipient mouse, iBAT derived from EB mice exhibited higher thermogenic capacity than iBAT from MF mice (Figure 3B,C). As a control, bilateral iBAT transplants from EB mice showed comparable thermogenic capacity (Figure S3A, Supporting Information). Anatomical analysis confirmed successful transplantation of iBAT from both EB and MF mice (Figure 3D). Notably, the iBAT from MF mice visually resembled white fat (Figure 3D). We also assessed vascularization and sympathetic innervation by examining CD31 and TH expression. No significant differences in CD31 or TH density were observed between EB‐ and MF‐derived grafts (Figure S3B–D, Supporting Information). However, both markers showed reduced density in transplanted BAT compared with the host's endogenous BAT (Figure S3B–D, Supporting Information).

Histologically, transplanted iBAT from EB mice retained characteristic brown adipose tissue properties, while transplanted iBAT from MF mice appeared more similar to white adipose tissue (WAT), exhibiting reduced UCP1 expression and larger lipid droplets (Figure 3E–H). To assess the duration of function in transplanted iBAT, we conducted a follow‐up over 12 weeks. Under 4 °C cold exposure, we observed a gradual decline in thermogenic capacity in the bilateral scapular region as transplantation time increased (Figure S3E,F, Supporting Information). However, iBAT derived from EB mice maintained higher thermogenic capacity than iBAT from MF mice throughout the 12‐week period (Figure S3E,F, Supporting Information).

To assess the effect of transplanted iBAT on the overall metabolism of recipient mice, we transplanted MF mice with iBAT derived from EB and MF mice, respectively, and subjected them to an HFD challenge (Figure 3I). As expected, mice receiving iBAT from EB mice gained weight more slowly than those receiving iBAT from MF mice, and showed improved glucose tolerance and insulin sensitivity (Figure 3K–N). We also measured EE at different time points during the HFD feeding and found that mice receiving iBAT from EB mice exhibited higher EE at both 4 and 12 weeks post‐transplant compared to those receiving iBAT from MF mice (Figure 3O,P). Notably, at 12 weeks, the EE difference between the two groups narrowed. Further, by 16 weeks, the EE difference between the two groups disappeared (Figure S3G, Supporting Information). Additionally, mice receiving iBAT from EB mice exhibited lower respiratory exchange ratio (RER) values, indicating greater fatty acid utilization (Figure S3H, Supporting Information).

To provide direct evidence that BAT plays a critical role in mediating the metabolic benefits of breastfeeding, we performed surgical BAT ablation in EB and MF mice after weaning. Under HFD conditions, body weight gain did not differ between EB and MF groups. However, BAT removal largely abolished the metabolic advantages of EB, including reduced fat mass and elevated EE (Figure 3Q,R; Figure S3I,J, Supporting Information). Although EB mice still exhibited modest improvements in GTT and ITT compared with MF mice, these effects were substantially attenuated relative to intact animals (Figure 3S–V).

### 2.4 AMPK Plays a Key Role in Triggering Thermogenic Memory

**Figure 4:**
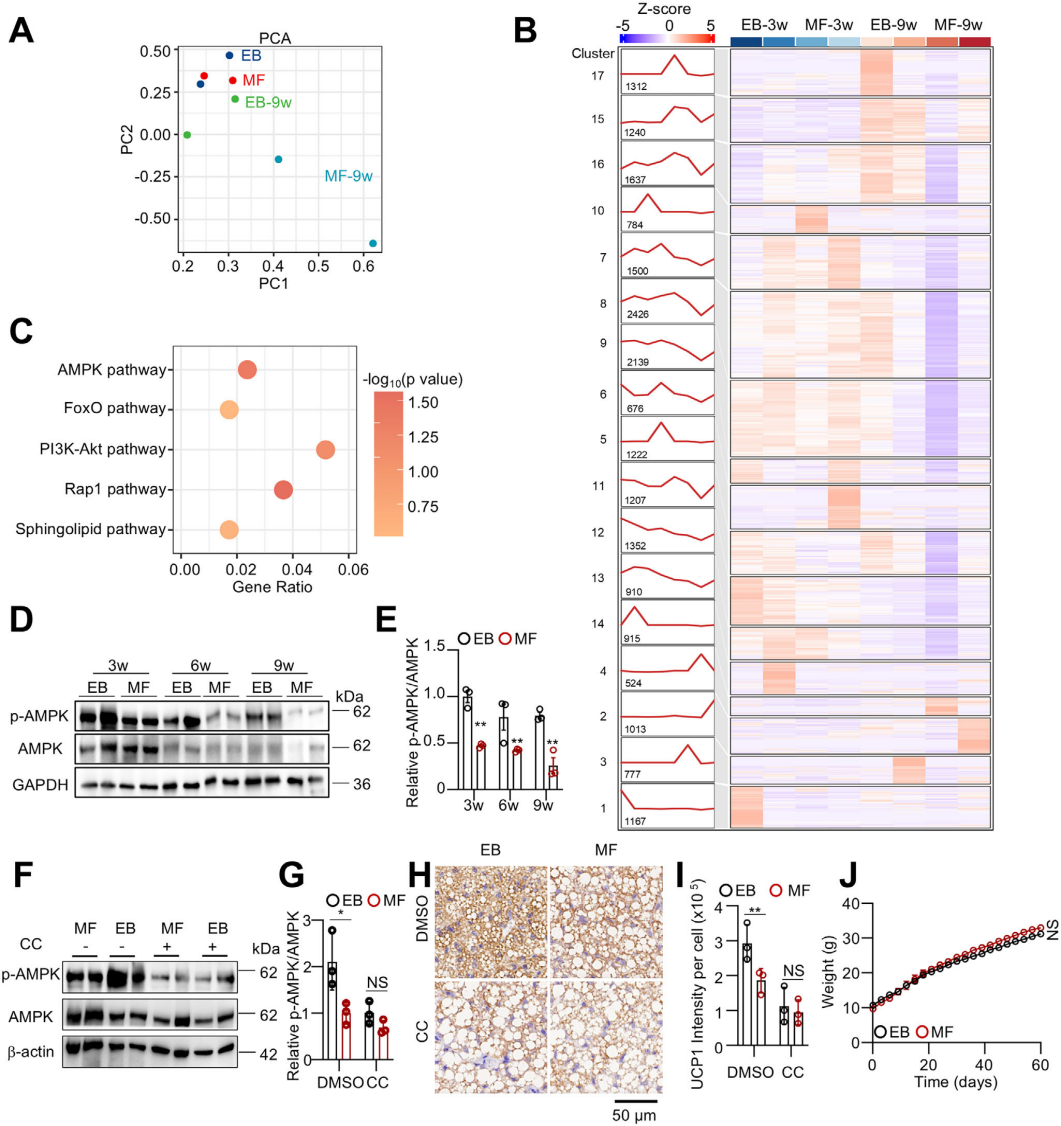
AMPK plays a key role in establishing thermogenic programming. A, B) Transcriptomic analysis was performed on BAT from EB and MF male mice at 3 and 9 weeks of age. Principal component analysis (PCA) (A) and gene expression patterns analysis (B) of RNA‐seq data were performed. C) KEGG pathway analysis was conducted for Cluster 11 genes. D, E) Western blot analysis (D) and quantification (E) were performed to assess AMPK phosphorylation changes in iBAT from EB and MF mice at different ages. (n = 3). F, G) AMPK phosphorylation in iBAT following Compound C (CC) treatment (5 mg/kg, subcutaneous injection, three times per week for one week) was assessed by Western blot analysis. (n = 3). H, I) Immunohistochemistry (IHC) analysis of UCP1 was performed, and the UCP1‐positive area was quantified. (n = 3). J) After CC treatment, EB and MF male mice were fed a 45% HFD, and body weight was monitored. (n = 3). Data are presented as mean ± SEM. Statistical significance was determined using a two‐way ANOVA (J) or a two‐tailed Student's *t*‐test for all other figures (^*^ *p* < 0.05; ^**^ *p* < 0.01; ^***^ *p* < 0.001).

To identify the key regulator of thermogenic memory in BAT, we conducted RNA sequencing (RNA‐seq) on iBAT samples from EB and MF mice at two time points: immediately after weaning (3 weeks old) and after 6 weeks on a chow diet post‐weaning (9 weeks old). Principal component analysis (PCA) revealed that, compared to MF mice, the gene expression profiles of EB mice were more similar at both 3 and 9 weeks (**Figure** **4**A).

Among all expressed genes, 17 clusters with distinct expression patterns across the four groups were identified (Figure 4B). Notably, the expression trend of cluster 11—a group of 1207 genes highly expressed in EB mice compared to MF mice—stood out. Even six weeks post‐weaning, this cluster maintained relatively higher expression levels in EB mice (EB‐9w) compared to age‐matched MF mice (MF‐9w) (Figure 4B). Transcription factor clustering analysis identified Pparγ, Pgc1α, Jun, and Nf‐κb as key transcription factors significantly associated with Cluster 11 genes. Furthermore, the mitochondrial thermogenesis‐related genes Ucp1 and Cidea are regulated by this transcriptional network (Figure S4A, Supporting Information). Biological process (BP) analysis identified peptidyl‐amino acid modification and protein maturation as top‐ranked processes in cluster 11 (Figure S4B, Supporting Information). Further Kyoto Encyclopedia of Genes and Genomes (KEGG) pathway enrichment of cluster 11 genes revealed significant involvement in the AMPK, FoxO, PI3K‐Akt, Rap1, and sphingolipid signaling pathways (Figure 4C). Notably, the catabolic process, highlighted in the BP analysis, is closely regulated by the AMPK signaling pathway. We then measured AMPK activity in iBAT during lactation and after weaning. As expected, phosphorylated AMPK (p‐AMPK) levels were higher in EB mice than in MF mice (Figure 4D,E). Although p‐AMPK levels decreased in iBAT of both groups post‐weaning, they remained significantly higher in EB mice, with this difference observable up to nine weeks of age (Figure 4D,E).

To investigate the role of AMPK in exclusive breastfeeding‐induced BAT activity, we administered the AMPK inhibitor Compound C (CC) at 5 mg kg^−1^ via subcutaneous injection into the interscapular region during the final week of lactation (weeks 2–3). CC treatment completely abolished the increase in AMPK phosphorylation induced by exclusive breastfeeding (Figure 4F,G). It also disrupted the metabolic benefits observed in EB mice, including elevated UCP1 expression, improved lipid droplet morphology, and enhanced thermogenesis (Figure 4H,I; Figure S4C–F, Supporting Information). Additionally, following an HFD challenge after weaning, no significant difference in weight gain was observed between EB and MF mice (Figure 4J).

### 2.5 Breast Milk‐Derived Extracellular Vesicles Promote AMPK Activation and BAT Thermogenesis

**Figure 5:**
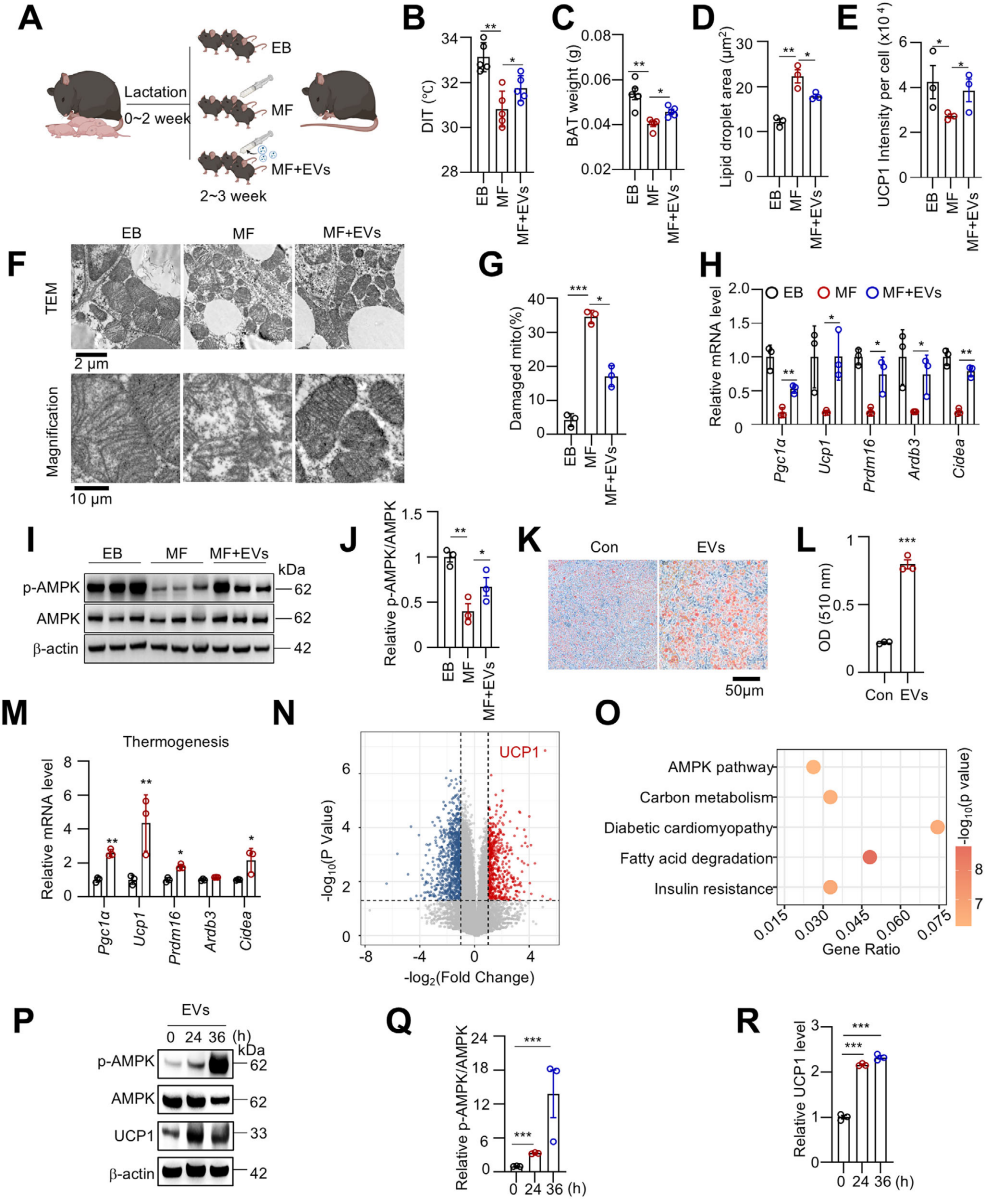
Breast milk‐derived extracellular vesicles promote AMPK activation and BAT thermogenesis. A) Schematic diagram of the animal experimental model. Two‐week‐old MF male mice were fed artificial milk formula supplemented with EVs daily for one week. B) DIT measurement in EB and MF mice (3 weeks old) after 1 h of cold exposure at 4 °C. (n = 5). C) iBAT weight in EB and MF mice. (n = 5). D) Quantification of lipid droplet size in iBAT based on H&E staining. (n = 3). E) Quantification of UCP1 IHC in iBAT. (n = 3). F, G) TEM images of iBAT showing mitochondrial morphology (F) and quantification of damaged mitochondria percentage (G). (n = 3). H) qRT‐PCR analysis of thermogenesis‐related gene expression in iBAT. (n = 3). I, J) Western blot analysis (I) of AMPK phosphorylation in iBAT and its quantification (J). (n = 3). K, L) BSVs were treated with milk EVs every two days during adipogenic differentiation, followed by oil red O staining after 8 days. Absorbance at 510 nm was quantified. (n = 3). M) Expression of thermogenic genes in BSVs after EV treatment, analyzed by qRT‐PCR. (n = 3). N) RNA‐seq analysis of BSVs treated with milk EVs for 12 h. The volcano plot shows differentially expressed genes (fold change >2, P < 0.05), with upregulated genes in red and downregulated genes in blue. O) KEGG pathway clustering analysis of significantly upregulated genes. P–R) BSVs were treated with milk EVs for the indicated time, and AMPK phosphorylation and UCP1 protein levels were analyzed by Western blot (P) and quantified (Q, R). (n = 3). Data are presented as mean ± SEM. Statistical significance was determined using a two‐tailed Student's *t*‐test (^*^ *p* < 0.05, ^**^ *p* < 0.01, ^***^ *p* < 0.001).

Breast milk is rich in extracellular vesicles (EVs), which protect their cargo from degradation in the gastrointestinal tract and can cross biological barriers such as the intestinal mucosa, placenta, and blood‐brain barrier in humans.^[^ ^27^ ^]^ After isolating EVs and labeling them with DiR dye, we detected DiR signals in iBAT 6 h after oral administration, confirming the successful delivery of breast milk EVs to BAT (Figure S5A–D, Supporting Information). To investigate the role of milk EVs in the BAT function regulation of exclusive breastfeeding, two‐week‐old MF pups were supplemented with human milk EVs (MF+EVs) during formula feeding (**Figure** **5**A). As expected, EV supplementation partially restored iBAT thermogenesis and mass (Figure 5B,C). Histological analysis revealed that EV administration increased UCP1 expression and reduced lipid droplet size in iBAT (Figure 5D,E; Figure S5E, Supporting Information). Additionally, mitochondrial structure and morphology were improved, and the expression of thermogenic genes was enhanced (Figure 5F–H). Notably, impaired AMPK activity in MF mice was also partially rescued by EV intervention (Figure 5I,J).

To determine whether EVs directly affect brown adipocytes, brown stromal vascular cells (BSVs) were isolated. The addition of EVs enhanced adipogenesis and upregulated genes associated with thermogenesis, fatty acid oxidation, and oxidative phosphorylation (OXPHOS) (Figure 5K–M; Figure S5F–I, Supporting Information). To investigate the cellular processes triggered by breast milk EVs in brown adipocytes, RNA‐seq analysis was performed on BSVs treated with EVs for 12 h. This analysis identified 1441 genes that were significantly upregulated and 1565 genes that were significantly downregulated following EV treatment (fold change > 2, P < 0.05) (Figure 5N). Notably, UCP1 was the most significantly upregulated gene in response to EV treatment (Figure 5N). Furthermore, KEGG pathway analysis of the upregulated genes revealed strong enrichment in the AMPK pathway (Figure 5O). Consistently, EV treatment significantly increased AMPK phosphorylation and UCP1 expression in BSVs (Figure 5P–R).

### 2.6 MiRNA‐125a‐5p in Milk EVs Promotes AMPK Activation

**Figure 6:**
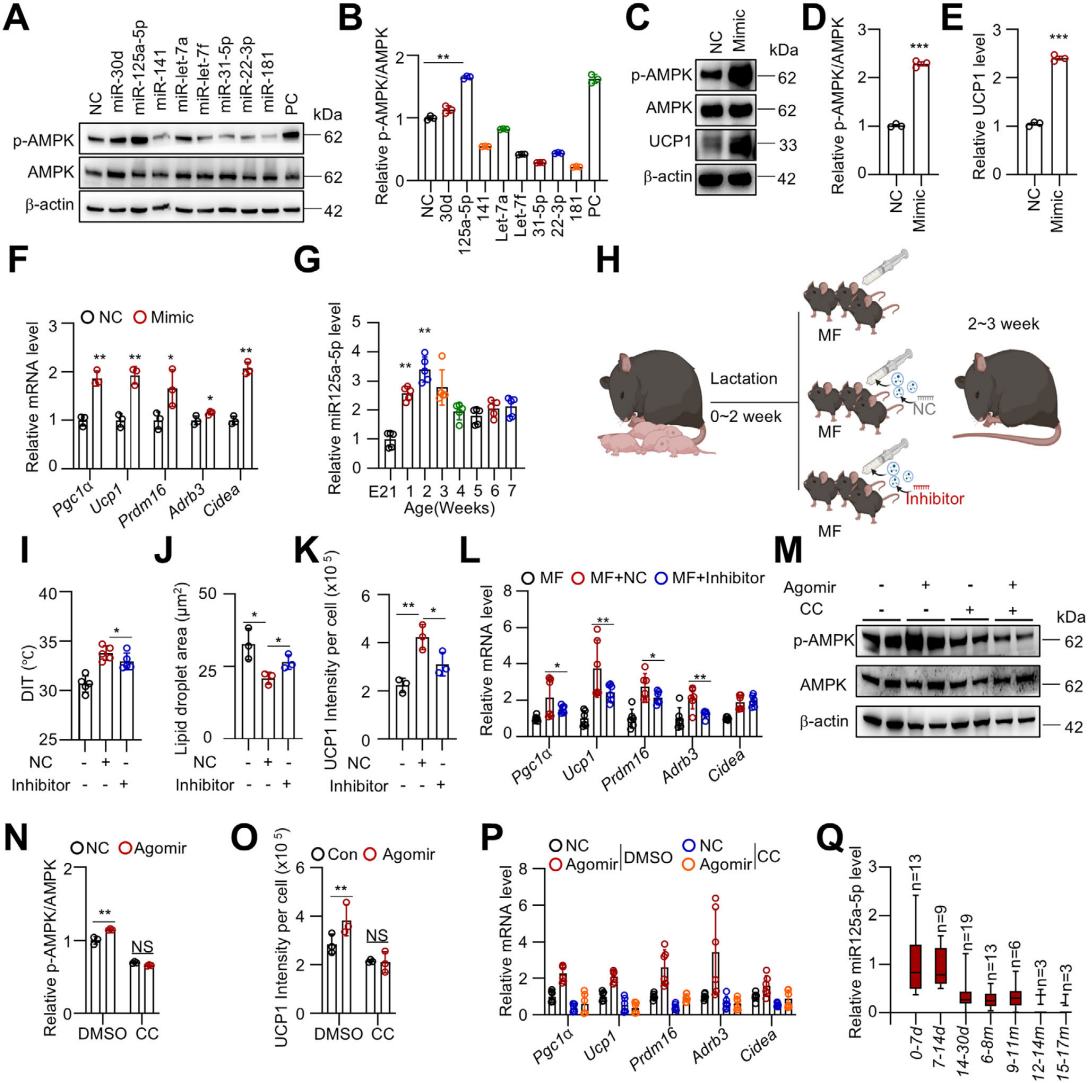
miRNA‐125a‐5p in milk EVs promotes AMPK activation. A, B) Screening of high‐abundance miRNAs in breast milk EVs. BSVs were transfected with indicated miRNA mimics, followed by Western blot analysis of AMPK phosphorylation and quantification. Positive control (PC), (n = 3). C–E) BSVs were transfected with miR‐125a‐5p, and Western blot analysis was performed to detect AMPK phosphorylation and UCP1 protein levels, followed by quantification. (n = 3). F) BSVs were transfected with miR‐125a‐5p mimic, and qRT‐PCR was performed to assess the expression levels of thermogenesis‐related genes. (n = 3). G) miR‐125a‐5p expression levels in iBAT were measured at embryonic day 21 (E21) and at different postpartum time points. (n = 5). H) After 2 weeks of breastfeeding, MF male mice were orally supplemented with formula milk and EVs. The EVs were pre‐loaded with either NC or miR‐125a‐5p inhibitor and administered daily for one week. I) DIT was measured in MF mice after 1 h of cold exposure at 4 °C. (n = 5). J, K) Histological analysis of lipid droplet size and UCP1 expression in iBAT. (n = 3). L) qRT‐PCR analysis of thermogenesis‐related gene expression in iBAT. (n = 6). M‐P) At 2 weeks of age, MF male mice were subcutaneously injected with CC and miR‐125a‐5p agomir in the interscapular region three times a week for one week. Western blot analysis of AMPK phosphorylation (M, N), IHC analysis of UCP1 expression in iBAT (O), and qRT‐PCR analysis of thermogenesis‐related gene expression in iBAT (P) were performed. Q) Expression levels of miR‐125a‐5p in breast milk EVs from normal postpartum women at different lactation stages. Data are presented as mean ± SEM. Statistical significance was determined using a two‐tailed Student's *t*‐test (^*^ *p* < 0.05, ^**^ *p* < 0.01, ^***^ *p* < 0.001).

Ingested milk EV RNA is bioavailable in infants and has been shown to regulate gene expression in tissues and influence the phenotype of the organism, representing a crucial mechanism for mother‐infant communication^[^ ^28^, ^29^ ^]^. Given the diversity and abundance of miRNAs in breast milk, we screened for highly abundant miRNAs in breast milk EVs.^[^ ^30^, ^31^ ^]^ Among these, miR‐141, miR‐31‐5p, and miR‐22‐3p were found to inhibit p‐AMPK, consistent with previous reports^[^ ^32^ ^]^ (**Figure** **6**A,B). In contrast, miR‐30d and miR‐125a‐5p elevated p‐AMPK levels (Figure 6A,B). The miR‐30d has been reported to promote AMPK phosphorylation.^[^ ^33^ ^]^ In addition, miR‐125a‐5p significantly increased UCP1 protein level and the expression of thermogenic genes (Figure 6C–F). It also upregulated genes associated with lipolysis, fatty acid oxidation (FAO), and oxidative phosphorylation (OXPHOS) (Figure S6A–D, Supporting Information). Notably, the expression of miR‐125a‐5p in iBAT significantly increased after birth, and declined after lactation (Figure 6G).

To investigate the role of miR‐125a‐5p in milk EV‐mediated iBAT thermogenesis, milk EVs were transfected with either miR‐125a‐5p inhibitor or a negative control (Figure 6H). qRT‐PCR analysis confirmed the successful encapsulation of the antagomir into these EVs (Figure S6E, Supporting Information). Additionally, EVs were transfected with Texas Red‐labeled siRNA and orally administered to mice. After 6 h, fluorescence was detected in iBAT, indicating that the modified EVs could effectively deliver miRNA to iBAT (Figure S6F,G, Supporting Information). MF mice were then orally administered the modified EVs along with formula milk once a day for one week (from weeks 2 to 3). Notably, the miR‐125a‐5p inhibitor diminished the beneficial effects of milk EVs on iBAT thermogenesis, lipid droplet morphology, and the expression of thermogenesis‐related genes (Figure 6I–L; Figure S6H–J, Supporting Information).

To directly validate the function of miR‐125a‐5p, a miR‐125a‐5p agomir was synthesized and subcutaneously injected into the interscapular region of mice. Administration of the miR‐125a‐5p agomir significantly increased DIT in response to cold exposure, UCP1 levels, thermogenesis‐related gene expression, and enhanced AMPK phosphorylation (Figure 6M–P; Figure S6K–O, Supporting Information). However, the promoting effect of the miR‐125a‐5p agomir on the BAT thermogenesis phenotype was abolished when the AMPK inhibitor CC was applied (Figure 6M–P; Figure S6K–O, Supporting Information). These findings suggest that miR‐125a‐5p plays a crucial role in EV‐mediated AMPK activation and iBAT thermogenesis. We then analyzed the variations in miR‐125a‐5p levels in human breast milk. As lactation progressed, the levels of miR‐125a‐5p in breast milk EVs gradually decreased and remained at a low level after six months (Figure 6Q).

### 2.7 miR‐125a‐5p Targets HIF1AN to Activate the AMPK Signal

**Figure 7:**
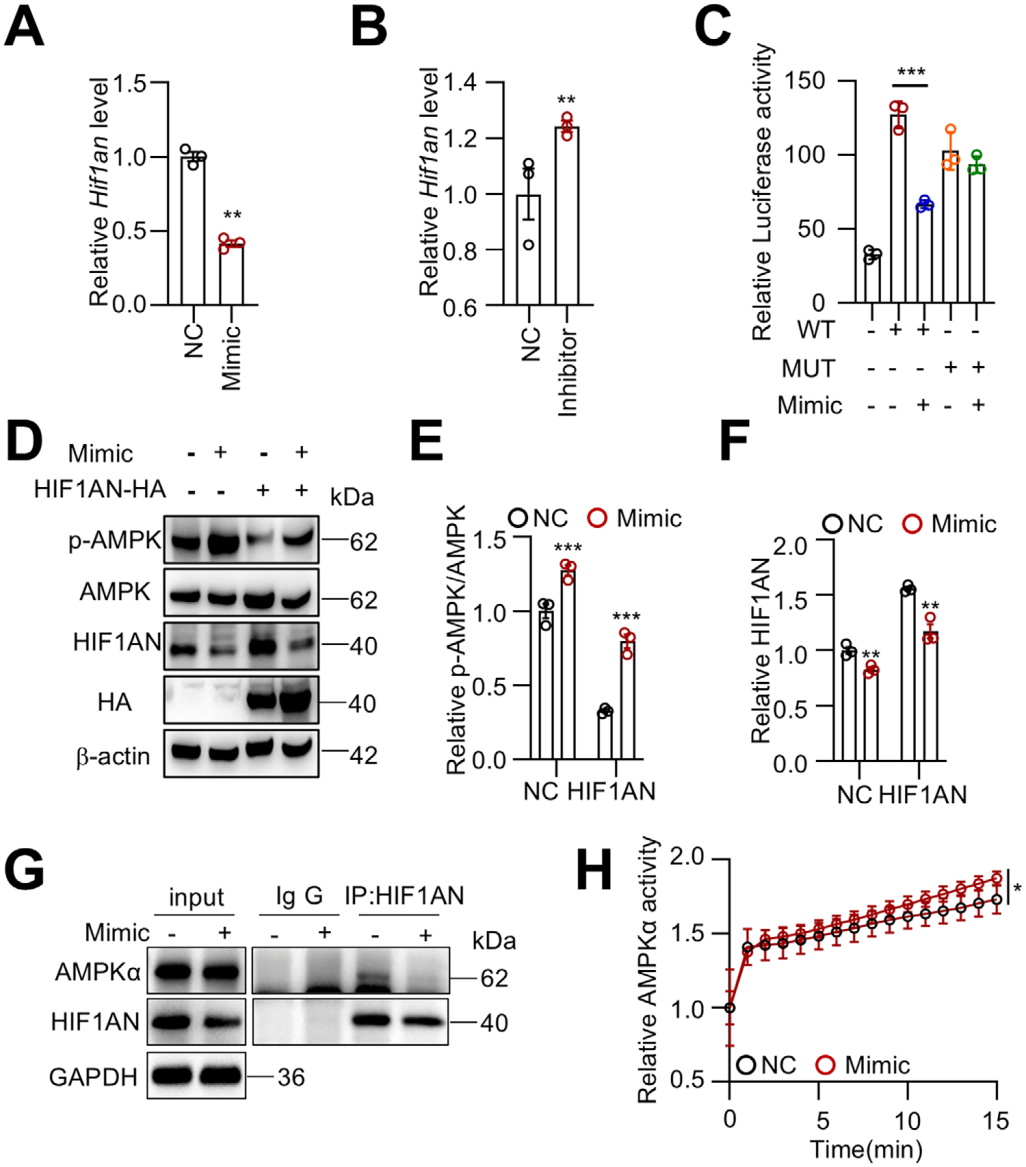
miR‐125a‐5p targets HIF1AN to activate the AMPK signal. A, B) BSVs were transfected with miR‐125a‐5p mimic (A) or inhibitor (B), and *Hif1an* expression was determined by qRT‐PCR. (n = 3). C) 293T cells were transfected with the dual luciferase reporter construct for 24 h, followed by transfection with miR‐125a‐5p mimic for an additional 24 h. Firefly luciferase activity was then measured and normalized to Renilla luciferase. (n = 3). D–F) BSVs were transfected with miR‐125a‐5p mimic and the HIF1AN expression construct for 48 h. AMPK phosphorylation and HIF1AN levels were assessed by Western blot and quantified. G) BSVs were transfected with miR‐125a‐5p mimic for 48 h, and endogenous HIF1AN was immunoprecipitated to analyze its interaction with AMPKα. H) BSVs were transfected with miR‐125a‐5p mimic for 48 h, and AMPKα was immunoprecipitated for subsequent kinase activity analysis. Data are presented as mean ± SEM. Statistical significance was determined using a two‐way ANOVA (H) or a two‐tailed Student's *t*‐test for all other figures (^*^ *p* < 0.05; ^**^ *p* < 0.01; ^***^ *p* < 0.001).

To identify miR‐125a‐5p targets, we used the prediction tools miRTarBase (mirtarbase.cuhk.edu.cn) and miRDB (mirdb.org). Among the top‐ranked predicted target genes in both datasets, HIF1AN (hypoxia‐inducible factor 1 alpha subunit inhibitor) caught our attention. HIF1AN is a hydroxylase that modifies the AMPKα1 subunit through hydroxylation, acting as a metabolic inhibitor for BAT thermogenesis.^[^ ^34^ ^]^ HIF1AN‐null mice have been reported to exhibit an elevated metabolic rate due to increased *Ucp1* and *Pgc1α* expression in iBAT.^[^ ^35^ ^]^ HIF1AN contains two highly conserved target sequences that perfectly match the 7‐mer seed region of miR‐125a‐5p (Figure S7A, Supporting Information), suggesting an evolutionarily conserved function of this miR‐125a‐5p/target pair. Additionally, the miR‐125a‐5p mimic decreased the mRNA level of *Hif1an*, while the inhibitor increased its level (**Figure** **7**A,B). Notably, *Hif1an* mRNA level was lower during lactation but significantly increased after weaning, displaying an inverse correlation with miR‐125a‐5p levels (Figure S7C, Supporting Information).

Most microRNAs inhibit their target gene expression by binding to the 3′ UTR, leading to mRNA degradation and protein translational suppression.^[^ ^36^ ^]^ To determine whether miR‐125a‐5p directly targets HIF1AN 3′ UTR, we generated reporter constructs containing luciferase cDNA linked to the 3′ UTR sequences of HIF1AN, as well as a mutant construct with four bases mutated to adenine (Figure S7B, Supporting Information). When the reporter constructs and miR‐125a‐5p were co‐transfected into 293T cells, miR‐125a‐5p significantly suppressed luciferase activity (Figure 7C). However, it had no effect on the mutated HIF1AN 3′ UTR‐induced signal (Figure 7C). Notably, overexpression of HIF1AN restored HIF1AN levels reduced by miR‐125a‐5p and simultaneously inhibited the AMPK activation induced by miR‐125a‐5p (Figure 7D–F). In addition, HIF1AN physically interacts with AMPKα1, and this interaction was markedly reduced after transfection with miR‐125a‐5p mimics (Figure 7G). Moreover, immunoprecipitation of AMPKα1 followed by activity assay revealed that AMPKα1 activity was significantly increased upon miR‐125a‐5p mimic transfection (Figure 7H).

### 2.8 miR‐125a‐5p/AMPK‐Induced αKG Promotes Brown Adipocyte Adipogenesis and Thermogenesis

**Figure 8:**
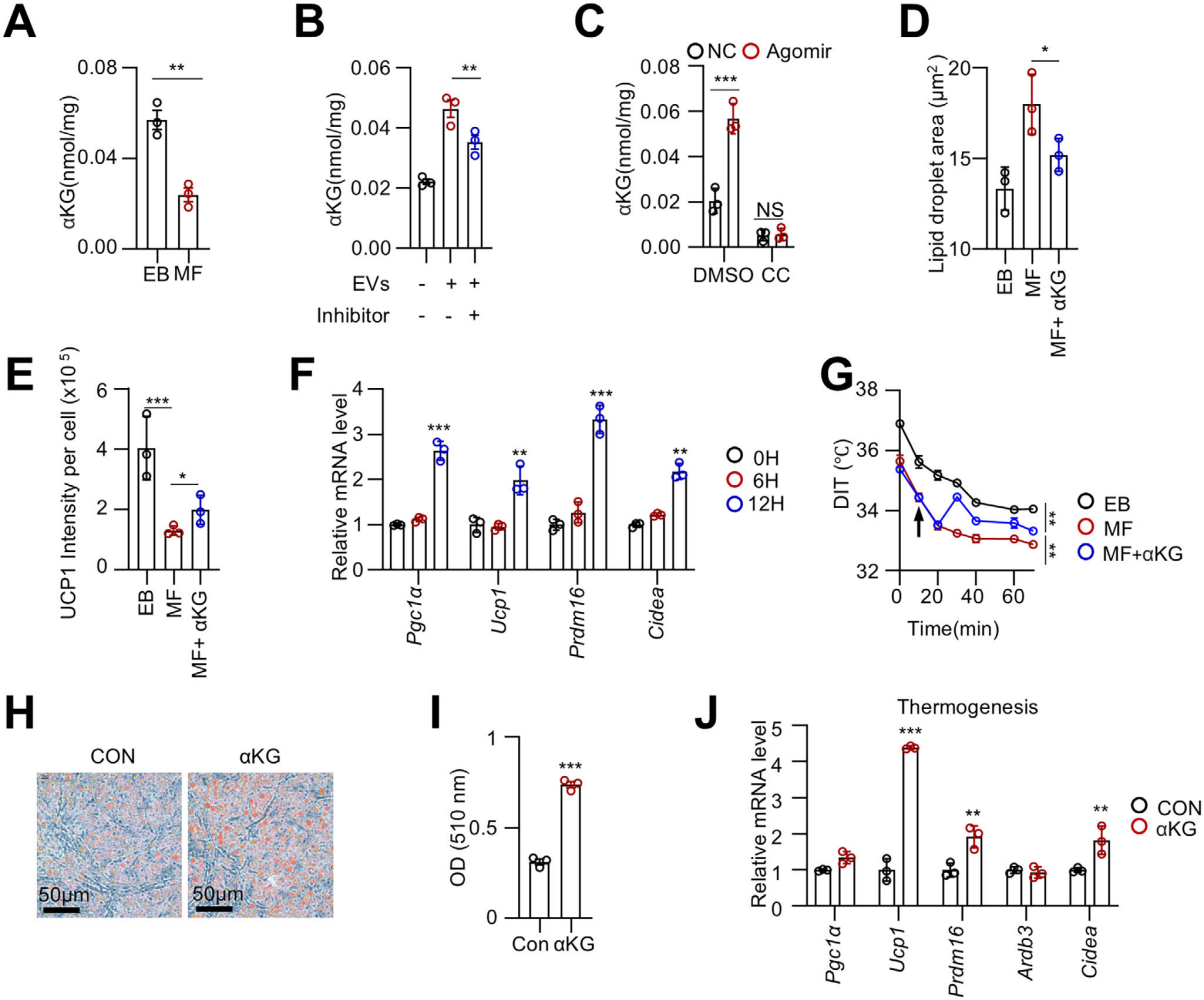
miR‐125a‐5p/AMPK‐induced αKG promotes brown adipocyte adipogenesis and thermogenesis. A) The αKG levels in iBAT of EB and MF male mice were measured. (n = 3). B) MF male mice were orally fed formula milk supplemented with milk EVs loaded with miR‐125a‐5p inhibitor, and αKG levels in iBAT were measured. (n = 3). C) MF male mice received interscapular subcutaneous injections of miR‐125a‐5p agomir and CC (5 mg/kg), and αKG levels in iBAT were assessed. (n = 3). D, E) MF male mice were injected subcutaneously with αKG (10 mg/kg) in the interscapular region three times per week for one week. H&E staining and UCP1 IHC were performed on iBAT tissue; the lipid droplet size and UCP1 levels were quantified. (n = 3). F) qRT‐PCR analysis of thermogenic gene expression in iBAT was performed after interscapular subcutaneous injections of αKG (10 mg/kg) for 12 h. (n = 3). G) Under 4 °C cold exposure, DIT was monitored in MF mice following interscapular subcutaneous αKG (10 mg/kg) injections. (n = 6). H, I) BSVs were induced for adipogenesis, and αKG (1 mM) was added every two days for a total of 8 days. Afterward, Oil Red O staining was performed, and absorbance at 510 nm (OD510) was measured. (n = 3). J) qRT‐PCR analysis of thermogenic gene expression in BSVs treated with αKG (1 mM) for 12 h. (n = 3). Data are presented as mean ± SEM. Statistical significance was determined using a two‐tailed Student's *t*‐test (^*^ *p* < 0.05, ^**^ *p* < 0.01, ^***^ *p* < 0.001).

We next examined α‐ketoglutarate (α‐KG), a key metabolite induced by AMPK activation. Indeed, αKG levels in BAT were significantly lower in the MF group compared to the EB group, but were partially restored by EV administration (**Figure** **8**A,B). EV treatment also significantly increased αKG levels in BSVs (Figure S8A, Supporting Information). However, this effect was diminished when EVs were transfected with the miR‐125a‐5p inhibitor (Figure 8B). Notably, the AMPK inhibitor CC abolished the miR‐125a‐5p‐induced increase in αKG levels in both iBAT and BSVs (Figure 8C; Figure S8B, Supporting Information). Additionally, ectopic expression of HIF1AN diminished the increased αKG levels induced by miR‐125a‐5p (Figure S8C, Supporting Information).

To investigate the role of αKG, subcutaneous injections were administered into the interscapular region of MF mice three times per week. αKG treatment enhanced the thermogenic phenotype (Figure 8D–F; Figure S8D–F, Supporting Information). Interestingly, DIT began to increase just 20 min after αKG administration in cold‐exposed mice (Figure 8G). In vitro, αKG directly enhanced adipogenesis in BSVs and promoted the expression of thermogenesis‐ and fatty acid oxidation‐related genes (Figure 8H–J; Figure S8G–J, Supporting Information).

### 2.9 αKG Could Rescue Impaired iBAT Function in MF Mice Following Weaning

**Figure 9:**
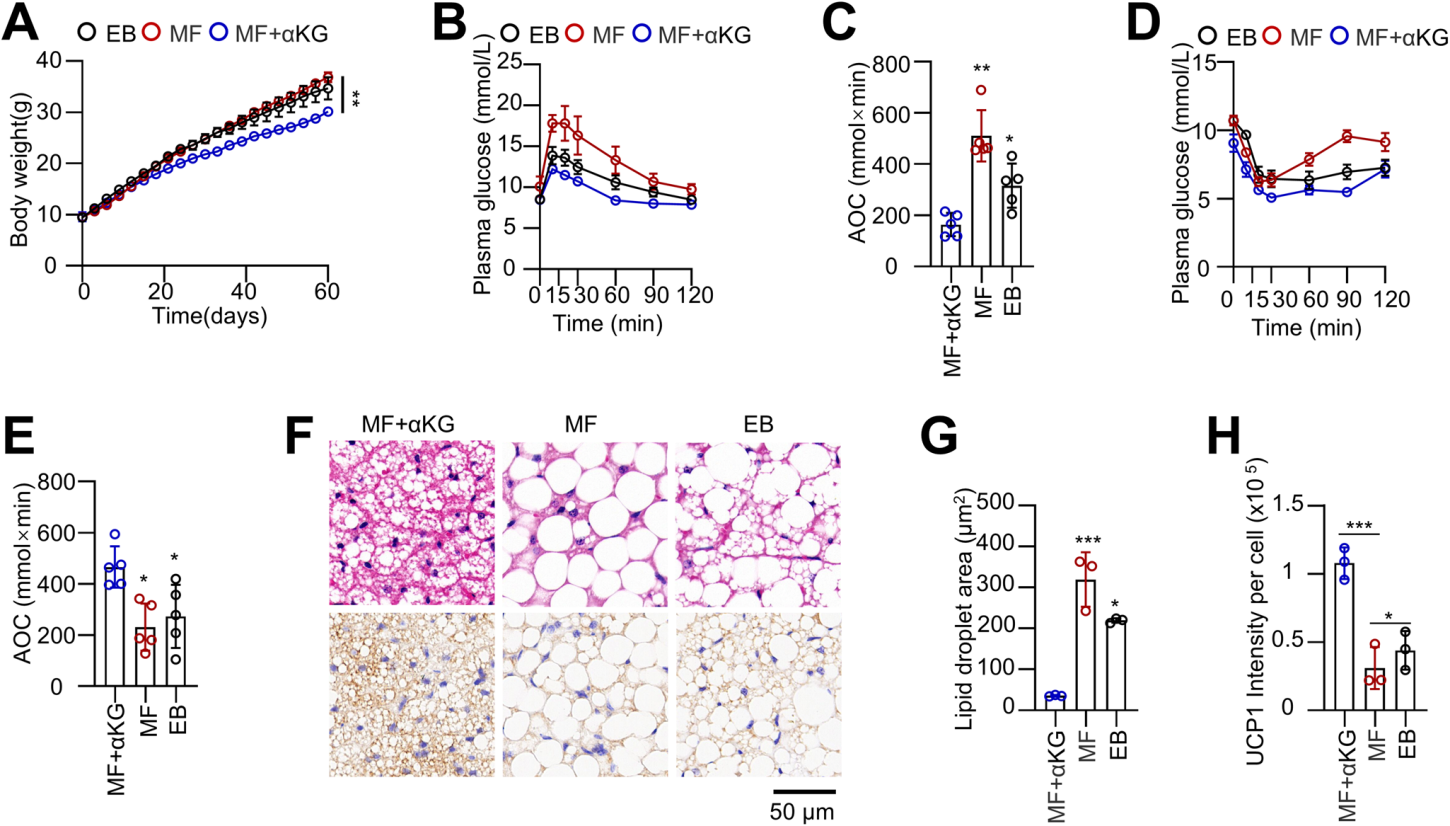
αKG could rescue impaired iBAT function in MF mice following weaning. A) Body weight curve of EB and MF male mice fed a 60% HFD and provided with 2% αKG in drinking water after weaning. (n = 5). B–E) GTT (B, C) and ITT (D, E) were performed after αKG intervention. (n = 5). F–H) H&E staining and UCP1 IHC analysis of iBAT in MF mice following αKG treatment. (n = 3). Data are presented as mean ± SEM. Statistical significance was determined by two‐way ANOVA for (A), and two‐tailed Student's *t*‐test for all other panels (^*^ *p* < 0.05, ^**^ *p* < 0.01, ^***^ *p* < 0.001).

Mothers often struggle to provide adequate breast milk to their offspring for various reasons. Therefore, whether αKG intervention can rescue this BAT function after weaning is an important and clinically relevant question. Water supplementation with αKG is well tolerated, and oral administration effectively increases circulating αKG levels.^[^ ^37^ ^]^ Thus, we provided MF mice with continuous αKG supplementation through drinking water after weaning. After ≈2 months of intervention, we found that αKG significantly reduced the body weight of MF mice without affecting their food intake (**Figure** **9**A; Figure S9A, Supporting Information). Additionally, αKG improved glucose tolerance and insulin sensitivity, and enhanced DIT under cold exposure (Figure 9B–E; Figure S9B,C, Supporting Information). Post‐intervention dissection revealed that αKG increased the UCP1 levels in iBAT and decreased the mass of iWAT and eWAT (Figure 9F–H; Figure S9D–G, Supporting Information).

## 3 Discussion

The mechanism underlying the long‐term protective effect of breastfeeding on the metabolic health of offspring remains largely unknown. In this study, we found that exclusive breastfeeding prevents HFD‐induced fat gain and glucose intolerance in offspring. Additionally, exclusive breastfeeding establishes metabolic memory in iBAT that persists into adulthood in mice. Further, miR‐125a‐5p, carried by breast milk EVs, mediates brown adipose development and thermogenesis through the HIF1AN/AMPK/αKG signaling axis. Notably, αKG appears to play a crucial role in this process, and post‐weaning αKG supplementation can rescue the abnormal BAT development caused by insufficient breastfeeding.

The potential long‐term protective effects of exclusive breastfeeding on energy balance and metabolic dysfunction in humans are not clear‐cut, as various confounding factors, such as socioeconomic conditions, maternal diet, and formula composition, influence both the mother and child over time.^[^ ^14^ ^]^ To address this, the mixed feeding protocol developed in this study provides an animal model with controlled experimental conditions, minimizing confounding factors and allowing for the identification and investigation of these long‐lasting effects and their underlying mechanisms. Here, we found that, compared to mixed‐fed mice, exclusively breastfed mice were more resistant to the HFD‐induced fat gain after weaning, and they also exhibited improved glucose tolerance and insulin sensitivity. This provides strong causal evidence at the animal level for previous cohort studies suggesting that exclusive breastfeeding can help prevent obesity and metabolic dysfunction in children.

Several physiological mechanisms have been hypothesized to link exclusive breastfeeding with a reduced risk of childhood obesity.^[^ ^14^ ^]^ These include differential appetite regulation between breastfed and bottle‐fed infants, the early protein hypothesis, the comparatively lower growth‐accelerating effect of breast milk, the role of leptin, and the association between obesity risk and variations in intestinal flora composition. Our findings showed no difference in food intake between EB and MF mice. The early protein hypothesis proposes that high protein intake from formula feeding promotes increased lipogenesis and fat cell development. Another putative mechanism indicates that formula feeding, compared to breastfeeding, accelerates growth during infancy.^[^ ^38^ ^]^ However, in the present study, no significant differences in lean body mass or body fat levels were observed between MF and EB mice at weaning, except for slight differences in brown fat mass.

Unlike formula milk, breast milk contains leptin, which appears to contribute to the protective effects of breastfeeding against obesity by suppressing food cravings and regulating calorie metabolism. Miralles et al.^[^ ^39^ ^]^ reported that leptin levels in breast milk during the first month of life may influence an infant's weight in the first and second years, particularly among mothers of normal weight. In our study, we found that MF mice had a slightly reduced serum leptin level; however, it did not affect their food intake level, and in the subsequent HFD challenge, their food intake was not significantly different from that of EB mice. Therefore, in our model, the effect on leptin levels may not be the decisive factor affecting the susceptibility of breastfeeding patterns to obesity in offspring.

Notably, our results showed that adding milk EVs to formula milk could partially restore iBAT thermogenesis in infants, while inhibition of miR‐125a‐5p within those EVs markedly attenuated this beneficial effect. These findings suggest that, in addition to miR‐125a‐5p, other components of breast milk EVs may also contribute to their thermogenic‐promoting effects. Breast milk is rich in various miRNAs, and previously reported EVs containing these miRNAs can reach systemic circulation, potentially impacting the epigenetic programming and development of various organs, including the liver, thymus, brain, pancreatic islets, and beige, brown, and white adipose tissues.^[^ ^40^ ^]^ However, most EVs and miRNAs are lost in formula milk,^[^ ^41^ ^]^ and we also found that miR125a‐5p is undetectable in formula (data not shown). Thus, miRNAs within milk EVs may serve as important players in the mechanism underlying the reduced risk of childhood obesity associated with exclusive breastfeeding.

Metabolic programming is a critical factor contributing to the development of obesity and the concurrent rise in related chronic diseases. As the primary source of nutrition for infants post‐neonatally, breastfeeding's effects on the reprogramming of energy balance during childhood and into adulthood are particularly emphasized. The perinatal period largely corresponds to the critical stages of brain maturation, neuronal differentiation, and active adipogenesis. In rodents, the differentiation of these systems primarily occurs during the last week of gestation, accelerates during early postnatal life, and remains active after weaning.^[^ ^42^ ^]^ The reprogramming effects of breastfeeding on neuronal signaling involved in metabolic regulation have been highlighted. Veronica et al. showed that extending breastfeeding from 3 to 4 weeks protects rats from obesity for up to 18 weeks. This effect is linked to the activation of hepatic FGF21‐mediated hypothalamic dopaminergic pathways, which enhance BAT thermogenesis.^[^ ^6^, ^43^ ^]^

Recent evidence suggests an important role for breast milk in the programming of infant adipose tissue. Yu et al. found that breast milk alkylglycerols contribute to the development of beige adipocytes and increase UCP1 expression in infant mice, a phenotype that could still be detected at five weeks of age.^[^ ^23^ ^]^ Our study found that exclusive breastfeeding reprograms AMPK activity in infant BAT, enhancing its development and thermogenesis. Notably, even after transplantation, the BAT from EB mice retains its potent thermogenic capacity, independent of the recipient mice. Conversely, surgical BAT ablation in EB and MF mice substantially attenuated the long‐term metabolic benefits of EB, but did not completely abolish these effects, suggesting that other metabolic tissues also contribute to mediating this process. Srinivasan et al. demonstrated that carbohydrate overnutrition during the suckling period alters early metabolic imprinting in pancreatic islet cells, resulting in immediate hyperinsulinemia that persists throughout life in rat pups fed a high‐carbohydrate formula.^[^ ^44^ ^]^ These findings indicate that lactation represents a critical postnatal window during which key metabolic regulatory organs can be reprogrammed through modifications in mother–infant interactions, including maternal metabolic factors and the nutritional environment. This period of responsiveness may shape metabolic memory and influence health later in life.

Interestingly, our findings also reveal sex‐specific differences in the metabolic effects of exclusive breastfeeding (Figure S2F–O, Supporting Information). Although both male and female offspring benefited from EB, the improvements in energy expenditure and glucose metabolism were more pronounced in males. This pattern is similar to previous reports showing that nutritional interventions during lactation exert sex‐dependent effects on offspring metabolism, with male offspring displaying more significant responses in serum triglycerides and glucose metabolism compared with females.^[^ ^45^ ^]^ These results highlight the importance of considering sex as a biological variable in early‐life nutritional programming.

AMPK has been proposed to regulate BAT thermogenic function indirectly by influencing the hypothalamus and the adrenergic nervous system in adults.^[^ ^46^, ^47^ ^]^ Recently, Yang et al. suggest that AMPK has a direct role in the epigenetic regulation of BAT development in neonatal mice through its impact on αKG.^[^ ^48^ ^]^ AMPK enhances the production of α‐KG, a key metabolite of the Krebs cycle, which promotes DNA demethylation of the PRDM16 promoter and thereby facilitates brown adipogenesis and thermogenesis.^[^ ^26^, ^49^, ^50^ ^]^ AMPK agonists administered during the early stage ameliorate impaired BAT development in weaning obese mice.^[^ ^48^ ^]^ Our data showed that pharmacological inhibition of AMPK during lactation significantly impairs brown adipogenesis and thermogenesis in EB mice. These findings highlight the critical role of AMPK in postnatal BAT development. αKG is regulated by AMPK and is required for TET‐mediated DNA demethylation of the Prdm16 promoter during early brown adipogenesis. Additionally, αKG can stimulate the release of adrenaline from the adrenal glands through the activation of 2‐oxoglutarate receptor 1 (OXGR1), which is expressed in the adrenal glands.^[^ ^51^ ^]^ This mechanism may explain the rapid increase in DIT following αKG administration.

Although environmental factors and maternal metabolic changes have been proposed to regulate AMPK activity in neonatal BAT, the physiological mechanisms governing this regulation remain elusive. HIF1AN is a hydroxylase that can directly hydroxylate AMPKα1, thereby inhibiting its activity. In whole‐body HIF1AN knockout mice, there was an increase in UCP1 expression in BAT and elevated energy expenditure.^[^ ^35^ ^]^ Zhang et al.^[^ ^34^ ^]^suggested that miR‐455 targets HIF1AN, leading to the activation of AMPKα1. This activation promotes the brown adipogenic program and enhances mitochondrial biogenesis. The role of HIF1AN in early BAT development has not been clarified. We uncovered a novel microRNA‐regulated signaling cascade in which miR‐125a‐5p activates the AMPK/αKG axis through targeting HIF1AN, acting as a metabolic trigger to initiate mitochondrial biogenesis and brown adipogenesis.

It is also important to consider that BAT development and thermogenic potential begin in utero, and that prenatal metabolic programming may influence postnatal responses to nutritional environments. During gestation, maternal nutritional status, endocrine factors (such as thyroid hormones and glucocorticoids), and placental nutrient transport play crucial roles in establishing fetal BAT progenitor pools and mitochondrial biogenesis.^[^ ^52^, ^53^, ^54^ ^]^ If BAT thermogenic capacity is already compromised during pregnancy, postnatal interventions, including breastfeeding, may have limited ability to fully restore its function. Although our current study primarily focuses on the lactation period, future investigations should explore how maternal metabolic perturbations during gestation affect the AMPK/αKG axis and whether prenatal programming interacts with lactation‐induced thermogenic memory. Integrating both prenatal and postnatal perspectives will provide a more comprehensive understanding of how early‐life nutrition shapes lifelong metabolic health.

A compelling question remains as to why AMPK activation persists at higher levels in EB mice over an extended period, particularly after weaning, a phenomenon that cannot be solely explained by breast milk EVs and miRNAs. One possible explanation is that AMPK activation during lactation promotes BAT development and establishes a robust thermogenic metabolic pattern. These characteristics may persist post‐weaning, as supported by the healthier BAT morphology observed in EB mice challenged with an HFD after weaning. Additionally, AKG‐mediated epigenetic markers may serve as a key biological basis for these long‐lasting effects. Additionally, it is worth noting that under 8 weeks of HFD feeding after weaning (Figure 2), there was no significant difference in body weight between EB and MF mice. However, in the iBAT transplantation experiment (Figure 3), a significant reduction in body weight was observed in the EB group after 12 weeks of HFD feeding. This suggests that the metabolic benefits conferred by EB on BAT may require a relatively longer period to manifest their impact on whole‐body weight regulation, even though improvements in white adipose tissue mass and overall metabolic profile are already evident at earlier time points.

Our study does have some limitations. The use of Compound C as a pharmacological inhibitor of AMPK introduces artificial elements that may constrain the interpretation of our findings. While we attempted to use Prkaa−/− mice, low breeding success posed significant challenges. We are currently working on constructing floxed mice to address this issue. In addition, we used milk EVs transfected with a miR‐125a‐5p inhibitor to probe its role in BAT, and fluorescence tracing showed that EVs distribute across multiple tissues. While BAT appears to be the primary responsive organ, as supported by BAT transplantation and ablation experiments, current methods cannot fully exclude potential effects of EV‐derived miR‐125a‐5p in other tissues. A systematic assessment of multiple metabolic organs will be essential to fully understand maternal milk–mediated metabolic regulation, representing an important avenue for future research.

In conclusion, we identified the miR‐125a‐5p/HIF1AN/AMPK/αKG axis as a key pathway linking exclusive breastfeeding to long‐term BAT activity and metabolic benefits in offspring. These findings provide new insights into preventing the risk of metabolic abnormalities in infants with insufficient breast milk.

## 4 Experimental Section

### Cell Culture and Treatment

Brown stromal vascular cells (BSVs) were isolated as previously described.^[^ ^26^ ^]^ In brief, 3‐week‐old male C57BL/6 mice that had been exclusively breastfed were sacrificed, and the harvested brown fat tissues were minced and digested in a 37 °C water bath with shaking for 1 h in a digestion buffer consisting of 50 mm HEPES (C0217, Beyotime, China), 2% bovine serum albumin (BSA) (V900933, Sigma–Aldrich, DEU), and 0.75 mg mL^−1^ collagenase B (11088807001, Sigma–Aldrich, DEU). The digested solution was then filtered through a 70 µm mesh strainer (352350, CORNING, USA), and the filtrate was centrifuged at 1000 rpm for 10 min. The cells enriched at the bottom formed the stromal vascular fraction, which was resuspended and cultured in high‐glucose Dulbecco's Modified Eagle Medium (DMEM) (Thermo Fisher, USA) containing 10% fetal bovine serum (FBS) (Thermo Fisher, USA) and 1% penicillin‐streptomycin (P/S) (V900929 Sigma–Aldrich, DEU). For adipogenic differentiation, the protocol followed a previously published method.^[^ ^55^ ^]^ Once confluent, BSVs were induced to differentiate into mature brown adipocytes by exposure to differentiation medium #1 for 2 days, consisting of DMEM, 10% FBS, 1% P/S, 20 mm HEPES (pH 7.4), 10 nm Triiodothyronine (S5726, Selleck, DEU), 0.1 mg mL^−1^ insulin (P3376, Beyotime, China), 0.5 mm isobutylmethylxanthine (IBMX) (ST1398, Beyotime, China), 1 µm dexamethasone (ST1254, Beyotime, China), and 0.125 mm indomethacin (HY‐14397, MedChemExpress, USA). The culture was then switched to differentiation medium #2 for six days, which included high‐glucose DMEM, 10% FBS, 1% P/S, 20 mm HEPES (pH 7.4), 0.1 mg mL^−1^ insulin, and 10 nm Triiodothyronine.

For in vitro treatments, differentiated BSVs were treated with EVs at a concentration of 2.5 × 10^8^ particles mL^−1^, or with α‐ketoglutarate (αKG) (328‐50‐7, MedChemExpress, USA) at 1 mm, and Compound C (866405‐64‐3, MedChemExpress, USA) at 20 µm for the indicated times, followed by harvesting for downstream analyses. For Oil Red O staining, differentiated adipocytes were fixed in 4% paraformaldehyde (BL539A, Biosharp, China) and stained with 0.5% Oil Red O (C0157S, Beyotime, China) to visualize lipid accumulation. For quantitative analysis of lipid content, the lipid droplets were eluted with isopropyl alcohol (I112011, Aladdin, China), and absorbance was measured spectrophotometrically (Thermo Scientific, USA) at 510 nm.

### Real‐Time Quantitative Reverse Transcription Polymerase Chain Reaction (qRT‐PCR)

Total RNA was extracted from cells using the EZ‐press RNA Purification Kit (B0004D, EZBioscience, USA) and from tissue using the EZ‐press RNA Purification Kit (RN4, EZBioscience, USA). DNA was removed by treatment with DNase I, and cDNA was synthesized using the HiScript III RT SuperMix kit (R323‐01, Vazyme, China). Quantification of cDNA was performed using the SYBR Green PCR Master Mix (Q311‐02/03, Vazyme, China) on the SLAN‐96S Real‐Time PCR machine (Shanghai HONGSHI Medical Tech, China), following the manufacturer's instructions. Transcript levels were normalized to Gapdh expression, and the primers used for qRT‐PCR are listed in Table S1 (Supporting Information).

### Western Blotting and Immunoprecipitation

For Western blot analysis, cells were lysed on ice in RIPA buffer (P0013B, Beyotime, China), and protein concentrations were determined using a BCA protein assay kit (E112‐01, Vazyme, China). The lysates were mixed with 5× protein loading buffer (LT101, Epizyme, China), and total protein extracts were separated on a polyacrylamide gel. Proteins were then transferred onto a nitrocellulose membrane. The following primary antibodies were used: rabbit anti‐P‐AMPK antibody (#2535, CST, USA, 1:1000 dilution), rabbit anti‐AMPK antibody (#5831, CST, USA, 1:1000 dilution), rabbit anti‐GAPDH antibody (#5174, CST, USA, 1:1000 dilution), rabbit anti‐β‐actin antibody (#4970, CST, USA, 1:1000 dilution), rabbit anti‐HA‐Tag antibody (#3724, CST, USA, 1:1000 dilution), rabbit anti‐GM130 antibody (11308‐1‐AP, Proteintech, China, 1:1000 dilution), rabbit anti‐CD9 antibody (20597‐1‐AP, Proteintech, China, 1:1000 dilution), rabbit anti‐UCP1 antibody (23673‐1‐AP, Proteintech, China, 1:1000 dilution), rabbit anti‐HIF1AN antibody (10646‐1‐AP, Proteintech, China, 1:1000 dilution). The following secondary antibodies were used: Goat Anti‐Rabbit IgG (BL003A, Biosharp, China, 1:10000 dilution). For detection, the membranes were incubated with chemiluminescence reagent (E422, Vazyme, China) and visualized using the Tanon 5200 Chemiluminescent Imaging System (Tanon, Shanghai, China).

For immunoprecipitation, BSVs were transfected with either miR‐125a‐5p mimic or a negative control mimic (NC) using Lipofectamine 2000 (Thermo Fisher Scientific, USA) according to the manufacturer's instructions. 48 h post‐transfection, cells were harvested and lysed in IP lysis buffer (50 mm Tris‐HCl, pH 7.5, 150 mm NaCl, 0.5% NP‐40) supplemented with protease and phosphatase inhibitors. A total of 1 mg of protein was incubated overnight at 4 °C with gentle rotation with 2 µg of anti‐HIF1AN antibody. Protein A/G magnetic beads (Beyotime, China) were then added and incubated for 2 h at 4 °C. Beads were washed six times with IP lysis buffer, and bound proteins were eluted in 2 × SDS sample buffer by heating at 95 °C for 5 min. Eluted proteins were subsequently analyzed by SDS‐PAGE and immunoblotting to detect interactions with AMPKα. For the AMPK activity assay, AMPKα was immunoprecipitated and eluted, and its activity was measured using the AMPK Kinase Activity Kit (Shanghai Jiwei Biological Technology Co., Ltd., G&VS50140.1.3) according to the manufacturer's instructions.

### Cell Transfection and Lentiviral Packaging

The miR‐125a‐5p mimic (miR10000135‐1‐5), inhibitor (miR20000135‐1‐5), miRNA NC (miR1N0000001‐1‐5), agomir (miR411627165150‐4‐5), and agomir NC (miR4N0000001‐4‐5) were purchased from RiboBio (Guangzhou, China). Cells were seeded in 6‐well plates and transfected with 200 nm of either the miRNA mimic or inhibitor using Lipofectamine 2000 (Thermo Fisher Scientific, USA). After 48 h of transfection, whole proteins or total RNA were harvested for subsequent Western blot or qRT‐PCR analysis.

Plasmids harboring HIF1AN were generated using the pHAGE vector. These plasmids were then subjected to lentivirus packaging. Lentivirus packaging was performed in HEK293T cells by transfection of transfer plasmid (pHAGE‐HIF1AN or pHAGE vector) together with packaging vectors (pCMV‐VSVG, pMDLg‐RRE (gag/pol), and pRSV‐REV) using polyethylenimine (PEI) (Beyotime, Shanghai, China). Virus particles were collected 48 h after transfection and then used for cell infection. To enhance the infection of each lentivirus, polybrene (5 µg mL^−1^) (H9268, Sigma–Aldrich, USA) was used for lentivirus infection. Following a 48 h incubation period, the efficiency of expression was evaluated.

### Dual Luciferase Reporter Experiment

The luciferase reporter construct pmirGLO‐HIF1AN 3′UTR (with part of the HIF1AN 3′UTR sequence removed and four potential binding regions retained; sequences are shown in Table S2, Supporting Information) or pmirGLO‐HIF1AN 3′UTR‐Mut was co‐transfected with the Renilla luciferase construct into HEK293T cells using Lipofectamine 2000 (Thermo Fisher, USA), following the manufacturer's protocol. After 24 h, the cells were further transfected with either the miR‐125a‐5p mimic or miRNA negative control (NC) for 24 h. A dual‐luciferase reporter assay was performed using the Firefly & Renilla Assay Kit (abs60341, Absin, China) according to the manufacturer's instructions. The treated cells were harvested and analyzed with the Fluoroskan Ascent FL fluorescence and chemiluminescence analyzer (Thermo, USA). Firefly luciferase activity was normalized to Renilla luciferase activity, and the data are presented as the ratio of Firefly to Renilla luciferase, with the control group set to 1.

### Human Milk Collection and Extracellular Vesicle (EV) Isolation

Breast milk samples were collected from women who gave birth at Wuxi Children's Hospital. Detailed participant information is provided in Table S3 (Supporting Information). All human studies were conducted in accordance with the principles of the Declaration of Helsinki. Informed consent was obtained from all participants, who were fully informed about the study's procedures and any potential risks. The study protocol and related experimental procedures were approved by the Ethics Committee of the Affiliated Children's Hospital of Jiangnan University (WXCH2023‐01‐030).

### Extracellular Vesicle (EV) Isolation, Characterization, Labeling, and miRNA Transfection

Breast milk samples were subjected to EV isolation using the Total Exosome Isolation Kit (4484450, Invitrogen, USA), according to the manufacturer's protocol. The isolated EVs were characterized using Western blotting (WB), transmission electron microscopy (TEM), and nanoparticle tracking analysis (NTA). RNA from the EVs was extracted with Trizol reagent (Invitrogen, USA) for further analysis.

For EV tracing, EVs were labeled with the fluorescent dye DiIC_18_(7) (D12731, Thermofisher, USA). The mixture was incubated at room temperature for 1 h to allow the dye to incorporate into the EV membranes. The labeled EVs were then administered to mice orally at a dose of 1 x 10^12^ particles kg^−1^. Fluorescent distribution was assessed 6 h post‐administration using a chemiluminescence imaging system (Tanon 5200, China).

miRNA encapsulation into EVs was performed as previously described.^[^ ^56^ ^]^ The isolated EVs were transfected with fluorescent siRNA control (Texas Red‐labeled siRNA), negative control, and artificial miRNA (miR‐125a‐5p inhibitor) using the Exo‐Fect Exosome Transfection Kit (System Biosciences, USA), following the manufacturer's instructions (≈10^10 particles were transfected with 20 nmol of miRNA). For the examination of miRNA encapsulation efficiency, synthetic miRNA (Cel‐miR‐39‐3p) was added to all samples and used as an exogenous control for data normalization. To trace the biodistribution of EVs following miRNA transfection, EVs encapsulating Texas Red–labeled siRNA were orally administered to mice. Fluorescent signals were examined at 6 and 12 h after gavage using a chemiluminescence imaging system.

### In Vivo Study

All animal experiments were conducted in accordance with the Guide for the Care and Use of Laboratory Animals published by the National Institutes of Health (NIH Publications No. 85–23, revised 1996) and received approval from the Ethics Committee of the Affiliated Children's Hospital of Jiangnan University (WXCH2024‐03‐062). C57BL/6 mice were obtained from Changzhou Cavens Laboratory Animal Bio Co., Ltd (Changzhou, China). The mice were housed in a temperature‐ and humidity‐controlled environment (23 °C ± 2 °C, 55% ± 5%) on a 12 h light/12 h dark cycle (8 am to 8 pm). Unless otherwise specified, the mice were maintained ad libitum on a standard mouse chow diet (SPF‐F02‐002, SPF Biotech, China) and had access to water at all times. Mice were anesthetized using isoflurane and euthanized by CO_2_ asphyxiation.

For artificial rearing, the procedure followed a previously reported method.^[^ ^23^ ^]^ After receiving breast milk for 2 weeks, male or female littermates were randomly divided into two groups: one group continued with exclusive breastfeeding (EB group), while the other group was introduced to mixed feeding (MF group). Littermates were separated from their mothers and placed in a 30 °C incubator for 4 h to induce fasting. They were then either sham‐fed (EB group) or given 100 µL g^−1^ of formula milk (MF group) before being returned to their mothers, where they continued to suckle maternal milk freely for the rest of the time. This feeding regimen was carried out once daily for one week until weaning. An artificial milk formula was developed following the methods described by Haidong Yu et al.^[^ ^23^ ^]^ Casein and whey protein served as the protein sources, while lactose was used as the carbohydrate. The fat content was adjusted to 16% by combining various oils. Detailed composition of the formula can be found in Table S4 (Supporting Information). To ensure thorough dissolution, the ingredients were mixed according to the protocol outlined by Yajima et al. using a sonicator (SCIENTZ‐IID, China). The milk was homogenized twice at high pressure (800–1000 bar) using an AH‐1500 high‐pressure homogenizer (ATS Engineering Limited, China). Nipples for artificial feeding were produced with some modifications based on a previous report.^[^ ^57^ ^]^ The nipples were fabricated from DPI 8400 polyurethane resin (hardness 50) using a mold, with the specific size parameters detailed in the supplemental materials. The nipple was attached to a 1 mL syringe, and artificial feeding was administered. For the high‐fat diet (HFD) challenge, after weaning, mice were fed a 45% or a 60% HFD (Research Diets, D12451) for the specified duration.

For EV intervention, 2‐week‐old MF mice were fed artificial milk formula supplemented with EVs (1 x 10^12^ particles/kg/day) for one week. The EVs were extracted from the breast milk of 20 women who delivered at full term, collected 1–6 months postpartum, fully mixed, and used as the intervention in the mice.

For drug treatment, 2‐week‐old mice received intrascapular subcutaneous injections of Compound C (5 mg kg^−1^) every two days (MedChemExpress, USA) for one week. Additionally, miR‐125a‐5p agomir was administered in the interscapular region three times a week at a dose of 2.5 nmol per mouse. For the αKG intervention during lactation, 10 mg kg^−1^ of αKG (MedChemExpress, USA) was administered via subcutaneous injection in the interscapular region, with the same volume of saline used as a control. For αKG intervention during the HFD challenge, mice were fed a 60% HFD (Research Diets, D12492). Simultaneously, they were provided with drinking water supplemented with 2% αKG.

For dorsal interscapular temperature (DIT) determination, mice were exposed to a cold environment at 4 °C, and the temperature of the interscapular region was measured using an infrared thermal imaging camera (K20, Hikvision, China). The mice were moved to a 30 °C environment to measure DIT in a thermoneutral condition. The core body temperature was assessed using a rectal probe connected to a digital thermometer (FT3400, DSJR, China).

### Histology and Immunohistochemistry

Paraffin‐embedded tissues were sectioned at 5 µm using a microtome and mounted on glass slides for hematoxylin and eosin (H&E) staining. For immunohistochemistry, epitope unmasking was performed by heat treatment in 10 mm sodium citrate buffer at 95 °C for 15 min. After antigen retrieval, the sections were preincubated with 5% non‐immune goat serum, followed by incubation with anti‐UCP1 antibody (23673‐1‐AP, Proteintech, China, 1:200 dilution) at room temperature for 2 h. The sections were then developed with 3,3′‐diaminobenzidine (DAB; Thermo Fisher, USA) for 5 min. Positive staining for UCP1 was quantified using ImageJ software (version 1.53 d).

### Energy Expenditure Measurements

The Energy Metabolism Monitoring System for animals (TOW Intelligent Technology, Shanghai, China) was utilized to evaluate metabolic parameters. The experiment was conducted under optimal conditions, maintaining a temperature of 25 °C with a 12 h light/dark cycle. For EE measurement under thermoneutrality (30 °C), mice were acclimated to 30 °C for 2 days prior to the experiment. Prior to starting the experiment, the system was calibrated with a standard gas mixture containing balanced ratios of O_2_, CO_2_, and nitrogen to ensure accurate measurements of metabolic parameters. Each animal was housed in an individual chamber equipped to measure O_2_ consumption, CO_2_ release, and the respiratory exchange ratio (RER). These parameters provided a detailed assessment of the animals' metabolic function.

### The Glucose Tolerance Test (GTT) and Insulin Tolerance Test (ITT)

Mice were fasted for 12 h for the glucose tolerance test (GTT) and 6 h for the insulin tolerance test (ITT). For the GTT, a 20% glucose solution (2 g kg^−1^ body weight) was administered intraperitoneally, while for the ITT, an insulin solution (0.75 U kg^−1^ body weight) was injected intraperitoneally. Blood samples were collected from the tail vein at 0, 15, 30, 60, 90, and 120 min to measure blood glucose levels using a Yuwell glucometer (230614, Yuwell, China). The area of the curve (AOC) for both the GTT and ITT was calculated for all treatment groups to quantify glucose tolerance and insulin sensitivity, following the method described in a previous report.^[^ ^58^ ^]^ In brief, AOC was calculated as the AUC minus the area under the baseline. The calculation was based on blood glucose concentration (mmol/L) multiplied by time (min); the resulting unit was expressed as mmol×min.

### Brown Fat Transplantation

Brown fat transplantation was conducted according to a previous report.^[^ ^48^ ^]^ In brief, BAT intended for transplantation was harvested from the interscapular region of 3‐week‐old mice (post‐weaning) and placed in pre‐warmed PBS prior to transplantation. Subsequently, 3‐week‐old recipient mice were anesthetized via isoflurane inhalation. ≈10 mg of donor BAT was carefully transplanted into the left and right subcutaneous regions, adjacent to the endogenous interscapular fat pads of the recipient mice, respectively. The skin was sutured using absorbable surgical sutures (3/8 2*6 6‐0, Ningbo Medical Needle Co., Ltd., China). After the procedure, the mice were fed either a standard chow diet or a high‐fat diet (HFD).

### Surgical iBAT Ablation

Surgical iBAT ablation was performed as previously described with minor modifications.^[^ ^59^ ^]^ Briefly, three‐week‐old EB and MF mice were anesthetized with isoflurane and positioned prone. The interscapular region was shaved and disinfected with 75% ethanol, and a 1–2 cm midline dorsal incision was made. Overlying white adipose tissue was bluntly dissected to expose the interscapular brown adipose depot, which was bilaterally and completely excised. Hemostasis was achieved with iodine swabs, and the incision was closed using 6‐0 absorbable sutures, followed by topical application of erythromycin ointment. Mice were recovered on a 37 °C warming pad and maintained on standard chow for one week to ensure complete wound healing before being switched to a 45% kcal high‐fat diet for the subsequent eight weeks.

### Electron Microscopy Analysis of Adipose Tissue

The protocols were based on previous reports.^[^ ^60^, ^61^ ^]^ For transmission electron microscopy (TEM), adipose tissues were fixed in a solution of 2.5% glutaraldehyde and 2.0% paraformaldehyde in 0.1 M sodium cacodylate buffer (pH 7.4) overnight at 4 °C. The tissues were then postfixed with 2.0% osmium tetroxide for 1 h at room temperature. Thin sections were subsequently stained with uranyl acetate and lead citrate before being examined using an HT7800/HT7700 electron microscope (Hitachi, Japan) for ultrastructural analysis. Damaged mitochondria were identified by loss of cristae and/or broken mitochondrial outer and/or inner membrane.

For scanning electron microscopy (SEM), adipose tissues were fixed in 3% vol/vol glutaraldehyde in PBS, followed by washing and postfixation for 1 h with 1% wt/vol OsO_4_ in PBS. After washing, the samples were dehydrated with gradient alcohol and dried using a critical point drying apparatus (K850, Quorum, UK). The dried samples were then mounted on conductive tape and sputter‐coated with gold before analysis using a JSM‐IT700HR scanning electron microscope (JEOL, Tokyo, Japan).

### RNA Sequencing (RNA‐Seq)

Brown tissue was extracted from 3‐week‐old male mice from both the EB and MF groups, as well as from 9‐week‐old EB and MF mice that had been fed a chow diet. BSVs were treated with EVs (2.5 × 10^8^ particles mL^−1^) for 12 h, after which the cells were collected for analysis. Total RNA was isolated using TRIzol (15596026CN, Invitrogen, USA), followed by library preparation according to Illumina standard instruction (VAHTS Universal V6 RNA‐seq Library Prep Kit for Illumina). High‐throughput RNA sequencing was performed by SHBIO Biotechnology Corporation (Shanghai, China) on the Illumina NovaSeq6000 (Illumina, USA). Differential expression from RNA‐seq experiments was assessed using fragments per kilobase of transcript per million mapped reads (FPKM), normalizing for gene length and sequencing depth. Genes with a *p*‐value < 0.05 in the comparisons were considered significantly differentially expressed genes (DEGs).

### Statistical Analysis

Statistical analysis and data visualization were performed using GraphPad Prism 8.0 (GraphPad Inc., San Diego, CA, USA). Data were analyzed using the Student's *t*‐test or two‐way ANOVA, depending on the experimental design. For energy expenditure analyses, analysis of covariance (ANCOVA) was conducted using the general linear model procedure in IBM SPSS Statistics, with group as a fixed factor and body weight as a covariate, to compare adjusted means. Results are presented as mean ± standard deviation (Mean ± SEM). Statistical significance was defined as *p* < 0.05. The number of experimental replicates or animals used is specified in the figure and respective figure legends.

## Conflict of Interest

The authors declare no conflict of interest.

## Author Contributions

N.W., A.Y., and X.Y. contributed equally to this work. Y.L., L.Z., and X.G. performed conceptualization and supervision. Y.L. performed instruction, methodology, and writing‐original draft preparation. N.W., A.Y., X.Y., M.W., J.Z., K.L., Y.H., M.C., Y.Z., X.Z., and Y.G. participated in investigation, visualization, software, and data curation.

## Supporting information



Supporting Information

## Data Availability

Data availability. All other data are included within the article or Supplementary Information or available from the authors on request.

## References

[1] K. Hu , A. E. Staiano , JAMA pediatrics 2022, 176, 1037. 35877133 10.1001/jamapediatrics.2022.2052 PMC9315946

[2] S. Caprio , N. Santoro , R. Weiss , Nat. metabolism 2020, 2, 223. 10.1038/s42255-020-0183-z PMC9425367 32694781

[3] A. Horesh , A. M. Tsur , A. Bardugo , G. Twig , Current Obesity Rep. 2021, 10, 301. 10.1007/s13679-021-00439-9 33950400

[4] D. J. P. Barker , J. American College Nutrit. 2004, 23, 588S. 10.1080/07315724.2004.10719428 15640511

[5] M. Lukaszewski , D. Eberlé , D. Vieau , C. Breton , American J. Physiol.‐Endocrinol. Metabol. 2013, 305, E1195. 10.1152/ajpendo.00231.2013 24045869

[6] V. Pena‐Leon , C. Folgueira , S. Barja‐Fernández , R. Pérez‐Lois , D. Silva , N. Lima , M. Martin , V. Heras , S. Martinez‐Martinez , P. Valero , C. Iglesias , Nat. Metabol. 2022, 4, 901. 10.1038/s42255-022-00602-z PMC9314260 35879461

[7] D. Pem , Adv. Practice Nurs. 2015, 1, 2573.

[8] R. C. Robertson , A. R. Manges , B. B. Finlay , A. J. Prendergast , Trends Microbiol. 2019, 27, 131. 30529020 10.1016/j.tim.2018.09.008

[9] N. F. Butte , M. G. Lopez‐Alarcon , C. Garza , Nutrient adequacy of exclusive breastfeeding for the term infant during the first six months of life, World Health Organization, Geneva Switzerland, 2002.

[10] K. D. Tambalis , S. Mourtakos , D. B. Panagiotakos , L. S. Sidossis , Breastfeeding Med. 2018, 13, 687. 10.1089/bfm.2018.0117 30411971

[11] J. W. Lee , M. Lee , J. Lee , Y. J. Kim , E. Ha , H. S. Kim , J. Korean Med. Sci. 2019, 34. 10.3346/jkms.2019.34.e85 PMC6417996 30886551

[12] M. Mantzorou , D. Papandreou , G. K. Vasios , E. Pavlidou , G. Antasouras , E. Psara , Z. Taha , E. Poulios , C. Giaginis , Nutrients 2022, 14, 3599. 36079855 10.3390/nu14173599 PMC9459704

[13] L. Twells , L. A. Newhook , Canadian J. Public Health 2010, 101, 36. 10.1007/BF03405559 PMC6974040 20364536

[14] S. N. Uwaezuoke , C. I. Eneh , I. K. Ndu , Clinical Med. Insights, Pediatrics 2017, 11, 1179556517690196. 28469518 10.1177/1179556517690196 PMC5398325

[15] J. Qiao , L. Dai , Q. Zhang , Y. Ouyang , J. Pediatric Nursing 2020, 53, 57. 10.1016/j.pedn.2020.04.024 32464422

[16] K. E. Bergmann , R. Bergmann , R. Von Kries , O. Böhm , R. Richter , J. Dudenhausen , U. Wahn , Int. J. Obesity 2003, 27, 162. 10.1038/sj.ijo.802200 12586995

[17] K. Huus , J. F. Ludvigsson , K. Enskär , J. Ludvigsson , BMC Pediatr. 2008, 8, 1. 18844983 10.1186/1471-2431-8-42 PMC2577650

[18] I. T. Bonomo , P. C. Lisboa , A. R. Pereira , M. C. F. Passos , E. G. de Moura , J. Endocrinol. 2007, 192, 339. 17283233 10.1677/joe.1.06952

[19] B. Gregg , L. Ellsworth , G. Pavela , K. Shah , P. K. Berger , E. Isganaitis , S. VanOmen , E. W. Demerath , D. A. Fields , Pediatric Obesity 2022, 17, 12892. 10.1111/ijpo.12892 PMC9177518 35060344

[20] X. Liang , Q. Yang , L. Zhang , J. W. Maricelli , B. D. Rodgers , M. Zhu , M. Du , Sci. Rep. 2016, 6, 34345. 27686741 10.1038/srep34345 PMC5043374

[21] T. Tsuduki , Y. Kitano , T. Honma , R. Kijima , I. Ikeda , J. Nutr. Sci. Vitaminol. 2013, 59, 384. 24418872 10.3177/jnsv.59.384

[22] M. Srinivasan , P. Mitrani , G. Sadhanandan , C. Dodds , S. Shbeir‐ElDika , S. Thamotharan , H. Ghanim , P. Dandona , S. U. Devaskar , M. S. Patel , J. Endocrinol. 2008, 197, 565. 18492820 10.1677/JOE-08-0021

[23] H. Yu , S. Dilbaz , J. Coßmann , A. C. Hoang , V. Diedrich , A. Herwig , A. Harauma , Y. Hoshi , T. Moriguchi , K. Landgraf , J. Clin. Invest. 2019, 129, 2485. 31081799 10.1172/JCI125646 PMC6546455

[24] J. S. Dyer , C. R. Rosenfeld , Seminars in Reproductive Medicine, Thieme Medical Publishers, Stuttgart, Germany 2011.

[25] Y. Wang , M. Gao , F. Zhu , X. Li , Y. Yang , Q. Yan , L. Jia , L. Xie , Z. Chen , Nat. Commun. 2020, 11, 1648. 32245957 10.1038/s41467-020-15488-2 PMC7125133

[26] Q. Yang , X. Liang , X. Sun , L. Zhang , X. Fu , C. J. Rogers , A. Berim , S. Zhang , S. Wang , B. Wang , M. Foretz , B. Viollet , D. R. Gang , B. D. Rodgers , M. J. Zhu , M. Du , Cell Metab. 2016, 24, 542. 27641099 10.1016/j.cmet.2016.08.010 PMC5061633

[27] J. Munir , A. Ngu , H. Wang , D. M. Ramirez , J. Zempleni , Pharm. Res. 2023, 40, 909. 36198923 10.1007/s11095-022-03404-w

[28] X. Jiang , L. You , Z. Zhang , X. Cui , H. Zhong , X. Sun , C. Ji , X. Chi , Front. Cell Developmental Biol. 2021, 9, 693534. 10.3389/fcell.2021.693534 PMC8267587 34249944

[29] C. Leroux , M. L. Chervet , J. B. German , Adv Nutr 2021, 12, 1625. 34022770 10.1093/advances/nmab059 PMC8483967

[30] E. Carrillo‐Lozano , F. Sebastián‐Valles , C. Knott‐Torcal , Nutrients 2020, 12, 3066. 33049923 10.3390/nu12103066 PMC7601398

[31] . F. Wu , X. Zhi , R. Xu , Z. Liang , F. Wang , X. Li , Y. Li , B. Sun , Ann. Transl. Med. 2020, 8, 1170. 33241019 10.21037/atm-20-5709 PMC7576086

[32] Z. Yousefi , M. Nourbakhsh , Z. Abdolvahabi , S. S. Ghorbanhosseini , Z. Hesari , S. Yarahmadi , S. Ezzati‐Mobasser , P. Seiri , M. Borji , R. Meshkani , J. Cell. Physiol. 2020, 235, 880. 31256424 10.1002/jcp.29002

[33] W. Li , G. Hou , J. Lv , F. Lin , G. Song , R. Li , Immunopharmacol. Immunotoxicol. 2021, 43, 431. 34157933 10.1080/08923973.2021.1933517

[34] H. Zhang , M. Guan , K. L. Townsend , T. L. Huang , D. An , X. Yan , R. Xue , T. J. Schulz , J. Winnay , M. Mori , EMBO Rep. 2015, 16, 1378. 26303948 10.15252/embr.201540837 PMC4766451

[35] N. Zhang , Z. Fu , S. Linke , J. Chicher , J. J. Gorman , D. Visk , G. G. Haddad , L. Poellinger , D. J. Peet , F. Powell , Cell Metab. 2010, 11, 364. 20399150 10.1016/j.cmet.2010.03.001 PMC2893150

[36] S. Djuranovic , A. Nahvi , R. Green , Science 2011, 331, 550. 21292970 10.1126/science.1191138 PMC3955125

[37] S. H. Naeini , L. Mavaddatiyan , Z. R. Kalkhoran , S. Taherkhani , M. Talkhabi , Exp. Gerontol. 2023, 175, 112154. 36934991 10.1016/j.exger.2023.112154

[38] P. Socha , R. Janas , A. Dobrzanska , B. Koletzko , I. Broekaert , D. Brasseur , A. Sengier , M. Giovannini , C. Agostoni , R. C. Monasterolo Insulin like growth factor regulation of body mass in breastfed and milk formula fed infants, Data from the EU childhood obesity programme. Early Nutrition and its Later Consequences, New Opportunities, Perinatal Programming of Adult Health—EC Supported Research, Springer, Berlin Germany, 2005. 10.1007/1-4020-3535-7_24 16137122

[39] O. Miralles , J. Sánchez , A. Palou , C. Picó , Obesity 2006, 14, 1371. 16988079 10.1038/oby.2006.155

[40] B. C. Melnik , W. Stremmel , R. Weiskirchen , S. M. John , G. Schmitz , Biomolecules 2021, 11, 851. 34200323 10.3390/biom11060851 PMC8228670

[41] A. Leiferman , J. Shu , B. Upadhyaya , J. Cui , J. Zempleni , J. Pediatric Gastroenterol. Nutrition 2019, 69, 235. 10.1097/MPG.0000000000002363 PMC6658346 31169664

[42] S. G. Bouret , S. J. Draper , R. B. Simerly , Science 2004, 304, 108. 15064420 10.1126/science.1095004

[43] C. Folgueira , D. Beiroa , B. Porteiro , M. Duquenne , E. Puighermanal , M. F. Fondevila , S. Barja‐Fernández , R. Gallego , R. Hernández‐Bautista , C. Castelao , Nat. Metabol. 2019, 1, 811. 10.1038/s42255-019-0099-7 PMC6774781 31579887

[44] M. Srinivasan , S. G. Laychock , D. J. Hill , M. S. Patel , Exp. Biol. Med. 2003, 228, 15. 10.1177/153537020322800102 12524468

[45] L. Ye , Q. Zhang , F. Xin , B. Cao , L. Qian , Y. Dong , Front. Cellular Infection Microbiol. 2021, 11, 621957. 10.3389/fcimb.2021.621957 PMC8017235 33816333

[46] P. B. M. de Morentin , I. González‐García , L. Martins , R. Lage , D. Fernandez‐Mallo , N. Martínez‐Sánchez , F. Ruíz‐Pino , J. Liu , D. A. Morgan , L. Pinilla , Cell Metab. 2014, 20, 41. 24856932 10.1016/j.cmet.2014.03.031 PMC4082097

[47] A. J. Whittle , S. Carobbio , L. Martins , M. Slawik , E. Hondares , M. J. Vázquez , D. Morgan , R. I. Csikasz , R. Gallego , S. Rodriguez‐Cuenca , Cell 2012, 149, 871. 22579288 10.1016/j.cell.2012.02.066 PMC3383997

[48] Q. Yang , X. Liang , X. Sun , L. Zhang , X. Fu , C. J. Rogers , A. Berim , S. Zhang , S. Wang , B. Wang , Cell Metab. 2016, 24, 542. 27641099 10.1016/j.cmet.2016.08.010 PMC5061633

[49] H. Wu , Y. Zhang , Cell 2014, 156, 45. 24439369 10.1016/j.cell.2013.12.019 PMC3938284

[50] H. Wu , A. C. D'Alessio , S. Ito , K. Xia , Z. Wang , K. Cui , K. Zhao , Y. E. Sun , Y. Zhang , Nature 2011, 473, 389. 21451524 10.1038/nature09934 PMC3539771

[51] . Y. Yuan , P. Xu , Q. Jiang , X. Cai , T. Wang , W. Peng , J. Sun , C. Zhu , C. Zhang , D. Yue , Z. He , J. Yang , Y. Zeng , M. Du , F. Zhang , L. Ibrahimi , S. Schaul , Y. Jiang , J. Wang , J. Sun , Q. Wang , L. Liu , S. Wang , L. Wang , X. Zhu , P. Gao , Q. Xi , C. Yin , F. Li , G. Xu , et al., EMBO J. 2020, 39, 103304. 10.15252/embj.2019103304 PMC7110140 32104923

[52] M. Symonds , M. Pope , D. Sharkey , H. Budge , Diabetologia 2012, 55, 1597. 22402988 10.1007/s00125-012-2505-5

[53] Y. Chen , Y. Hu , Q. Yang , J. S. Son , X. Liu , J. M. de Avila , M. Zhu , M. Du , Diabetes 2020, 69, 1662. 32409491 10.2337/db20-0009 PMC7372078

[54] R. Oelkrug , L. Harder , M. Pedaran , A. Hoffmann , B. Kolms , J. Inderhees , S. Gachkar , J. Resch , K. Johann , O. Jöhren , Nat. Commun. 2023, 14, 6742. 37875497 10.1038/s41467-023-42425-w PMC10597992

[55] M. Shao , J. Ishibashi , C. M. Kusminski , Q. A. Wang , C. Hepler , L. Vishvanath , K. A. MacPherson , S. B. Spurgin , K. Sun , W. L. Holland , Cell Metab. 2016, 23, 1167. 27238639 10.1016/j.cmet.2016.04.023 PMC5091077

[56] C. Castaño , S. Kalko , A. Novials , M. Párrizas , Proc. Natl. Acad. Sci. USA 2018, 115, 12158. 30429322 10.1073/pnas.1808855115 PMC6275521

[57] J. Hoshiba , Contemp. Top. Lab. Anim. Sci. 2004, 43, 50. 15174819

[58] S. Virtue , A. Vidal‐Puig , Nat. Metabol. 2021, 3, 883. 10.1038/s42255-021-00414-7 34117483

[59] Z. Piao , B. Zhai , X. Jiang , M. Dong , C. Yan , J. Lin , W. Jin , Biochem. Biophys. Res. Commun. 2018, 501, 807. 29775611 10.1016/j.bbrc.2018.05.089

[60] . A. Bartelt , O. T. Bruns , R. Reimer , H. Hohenberg , H. Ittrich , K. Peldschus , M. G. Kaul , U. I. Tromsdorf , H. Weller , C. Waurisch , Nat. Med. 2011, 17, 200. 21258337 10.1038/nm.2297

[61] M. J. Harms , J. Ishibashi , W. Wang , H. Lim , S. Goyama , T. Sato , M. Kurokawa , K. Won , P. Seale , Cell Metab. 2014, 19, 593. 24703692 10.1016/j.cmet.2014.03.007 PMC4012340

