# Supplementary material for: Exclusive Breastfeeding Drives AMPK‐Dependent Thermogenic Memory in BAT and Promotes Long‐Term Metabolic Benefits in Offspring
Source: Adv Sci (Weinh). 2025 Dec 19;13(12):e08956. doi: 10.1002/advs.202508956 (PMC12948208; doi:10.1002/advs.202508956)
Supplement: Supplementary file 1 — Supporting Information [file ADVS-13-e08956-s001.docx]

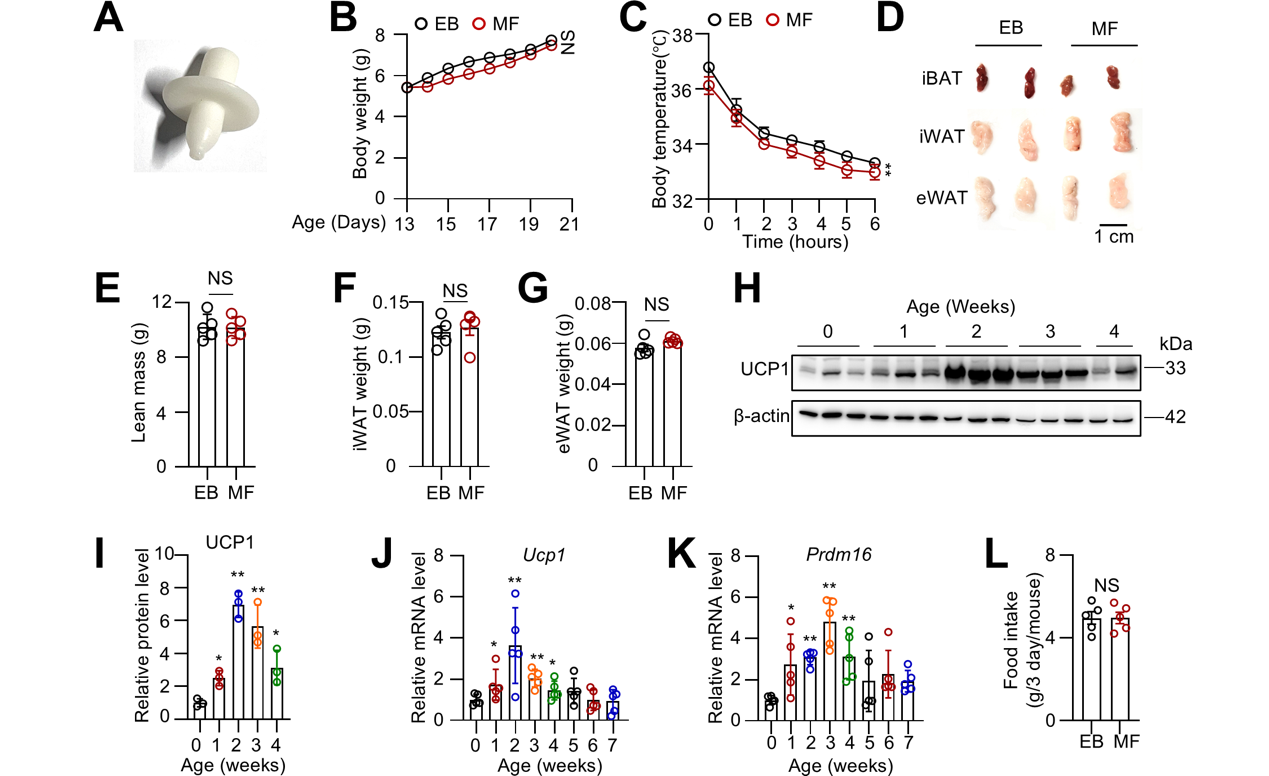


**Supplemental Figure 1. Body weight and adipose tissue composition in EB and MF mice**

A. The nipple used for artificial rearing.

B. Body weight of EB and MF male mice during artificial rearing at 2–3 weeks of age. (n=5)

C. Body temperature of EB and MF mice following 4°C cold exposure. (n=5)

D. Representative images of adipose tissue from different anatomical sites in EB and MF mice at 3 weeks of age.

E–G. Lean body mass (E), inguinal white adipose tissue (iWAT) (F), and epididymal white adipose tissue (eWAT) (G) in EB and MF mice. (n=5)

H, I. UCP1 protein (H) and quantification (I) in iBAT of male mice at different ages.

J, K. qRT-PCR analysis of *Ucp1* and *Prdm16* mRNA levels in iBAT at different ages. (n=5)

L. Food intake of EB and MF pups. (n=5)

Data are presented as mean ± SEM. Statistical significance was determined using a two-way ANOVA (B, C) or a two-tailed Student’s t-test (E-G, I-L) (*p < 0.05; **p < 0.01; ***p < 0.001).


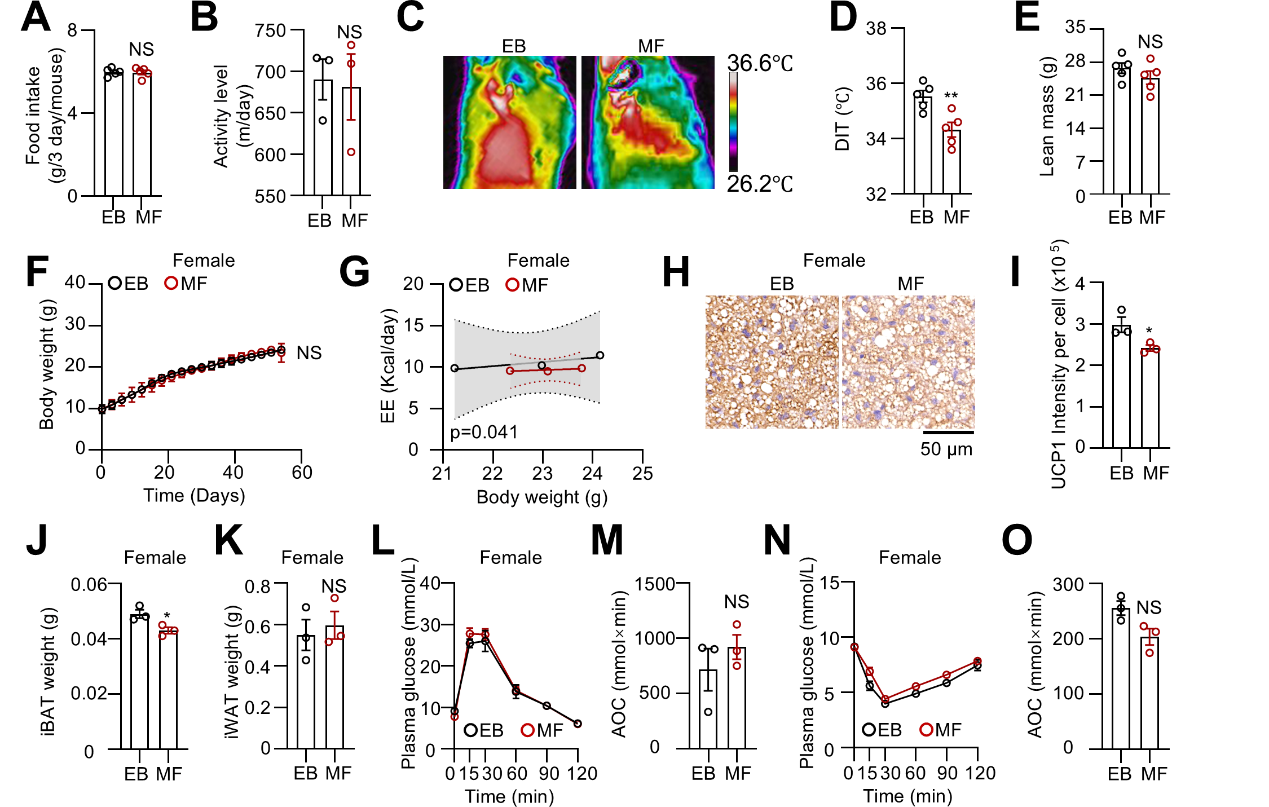


**Supplemental figure 2. Exclusive breastfeeding promotes metabolic health in response to a high-fat diet challenge**

A. Food intake of EB and MF male mice after HFD intervention. (n=5)

B. Locomotor activity in EB and MF mice following HFD intervention. (n=3)

C, D. The DIT in EB and MF mice under cold exposure was measured. (n=5)

E. The lean body mass of EB and MF mice after HFD treatment was assessed. (n=5)

F. Body weight of EB- and MF-treated female mice challenged with a 45% HFD (n=3).

G. Covariate analysis (ANCOVA) of energy expenditure (EE) versus body weight in female mice following HFD challenge (n=3).

H, I. UCP1 immunohistochemistry and quantification of iBAT in female mice (n=3).

J, K. The weight of iBAT and iWAT in female mice after HFD treatment was assessed (n=3).

L, M. Glucose tolerance test (GTT) and area of the curve (AOC) analysis in female mice following HFD. (n=3)
N, O. Insulin tolerance test (ITT) and AOC analysis in female mice after HFD (n=3).

Data are presented as mean ± SEM. Statistical significance was determined by two-way ANOVA for (F), ANCOVA with body weight as a covariate for energy expenditure analyses (G), and two-tailed Student’s t-test for all other panels (*p < 0.05, **p < 0.01, ***p < 0.001).


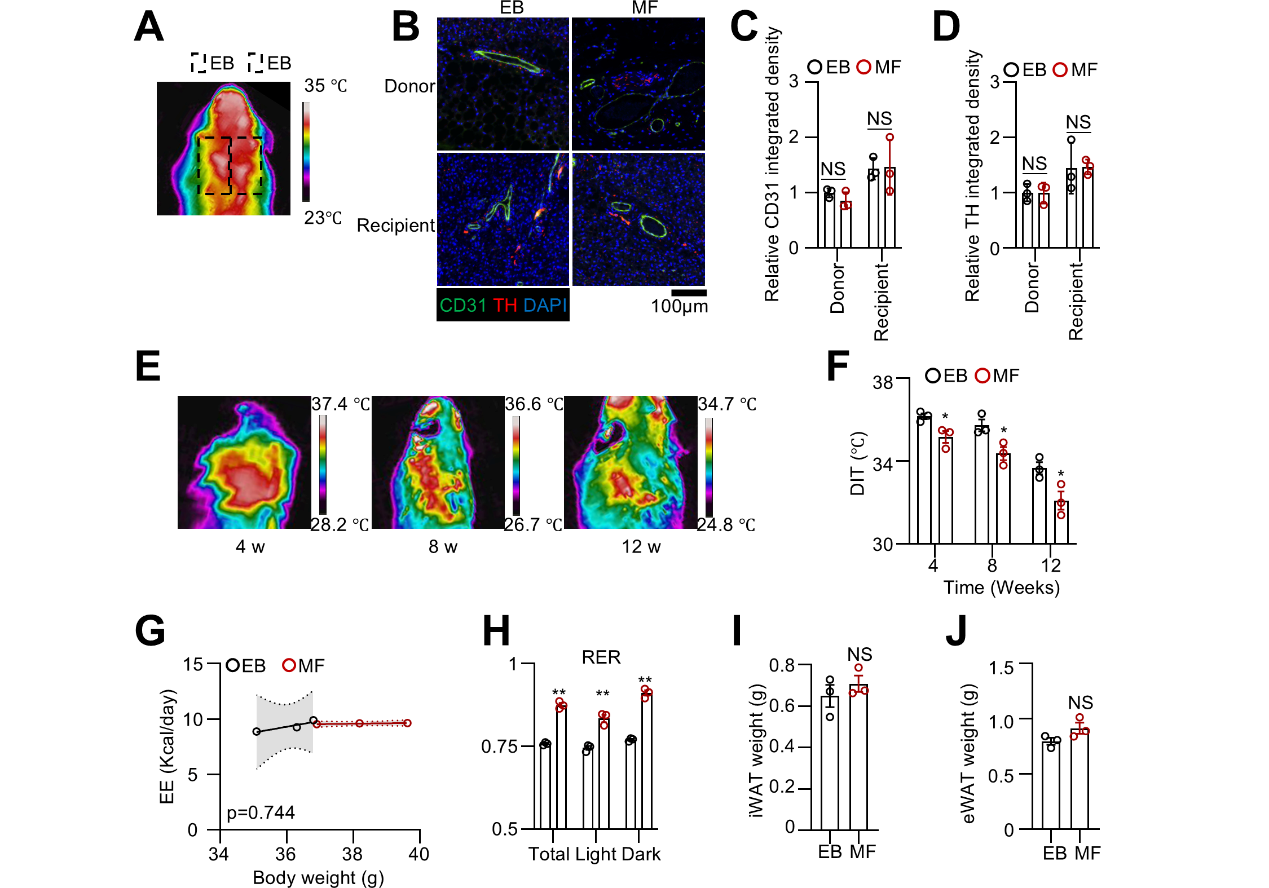


**Supplemental figure 3. Thermogenic programming shaped by Exclusive breastfeeding lasted 12 weeks post weaning**

A. Two 10 mg samples of iBAT were harvested from 3-week-old EB male mice and transplanted into each side of the scapular region of the same 3-week-old EB mice. The DIT under cold exposure was examined.

B-D. Immunofluorescence staining of iBAT sections from recipient mice was performed using antibodies against CD31 (green) to label endothelial cells and tyrosine hydroxylase (TH, red) to identify sympathetic innervation. Nuclei were counterstained with DAPI (blue). Scale bar = 100 μm.

E, F. At 4, 8, and 12 weeks after iBAT transplantation, the DIT was measured under 4°C cold stimulation. (n=3)

G. At week 16, energy expenditure (EE) was measured. (n=3)

H. At week 4, the respiratory exchange ratio (RER) was measured.

I, J. The weight of iWAT and eWAT in iBAT-removed male mice after HFD treatment was assessed (n=3).

Data are presented as mean ± SEM. Statistical significance was determined by ANCOVA with body weight as a covariate for energy expenditure analyses (G), and two-tailed Student’s t-test for all other panels (*p < 0.05, **p < 0.01, ***p < 0.001).


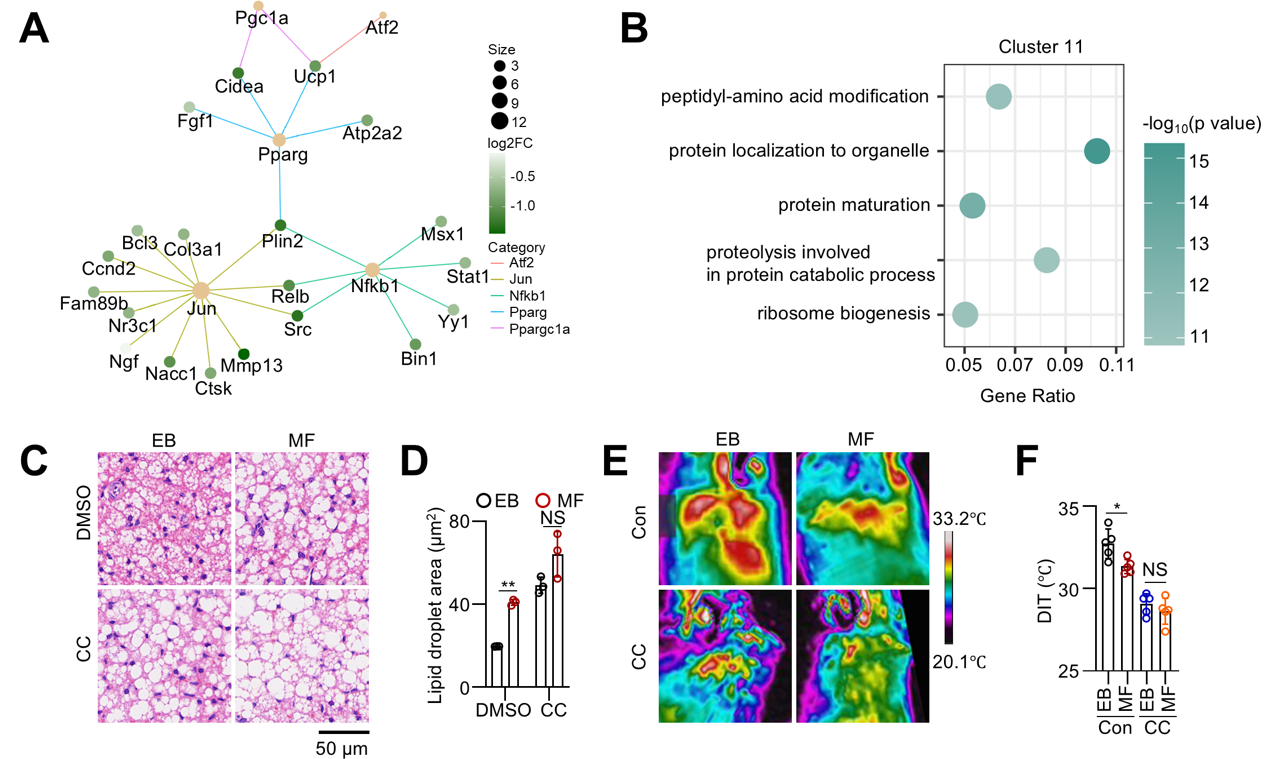


**Supplemental figure 4. Exclusive breastfeeding activates AMPK signal in BAT**

A. Transcription factor clustering analysis of Cluster 11 genes was performed using the TRRUST database.
B. Gene ontology (GO) analysis was conducted for Cluster 11 genes.
C, D. H&E staining of iBAT from EB and MF male mice following CC treatment.
E, F. DIT in EB and MF mice in response to 4°C cold exposure.

Data are presented as mean ± SEM. Statistical significance was determined using a two-tailed Student’s t-test (*p < 0.05, **p < 0.01, ***p < 0.001).


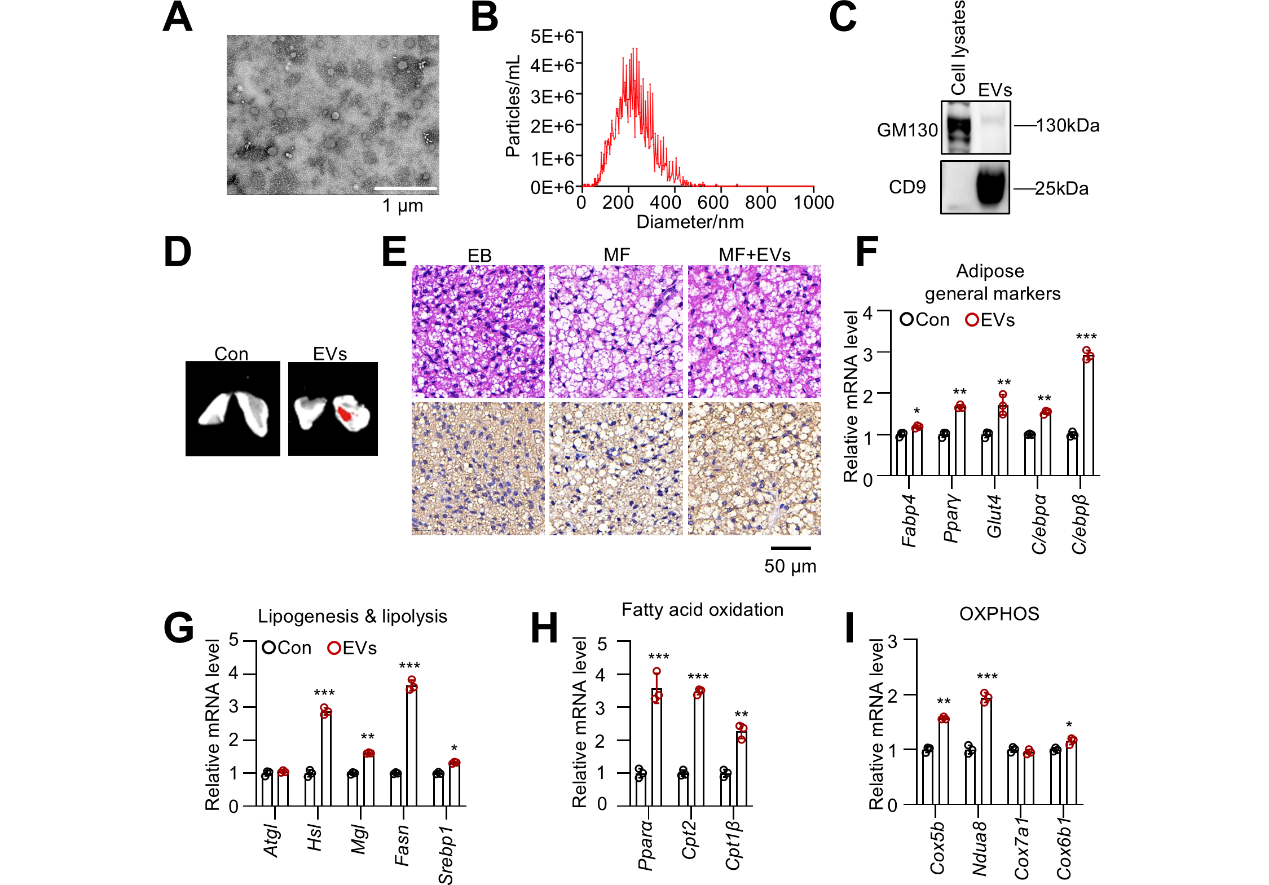


**Supplemental figure 5. Milk EVs promotes BAT thermogenesis**

A-C. Milk EVs were isolated and quality-checked using TEM (A), NTA (B), and Western blot analysis (C).
D. After labeling EVs with DiR dye (DilC18(7)), fluorescence signals in iBAT were detected 6 hours post oral administration.
E. MF mice were supplemented with EVs during daily formula feeding, and after one week, iBAT was analyzed by H&E staining and UCP1 IHC.
F-I. Expression levels of genes related to lipid metabolism in BSVs were measured by qRT-PCR. (n=3)

Data are presented as mean ± SEM. Statistical significance was determined using a two-tailed Student’s t-test (*p < 0.05, **p < 0.01, ***p < 0.001).


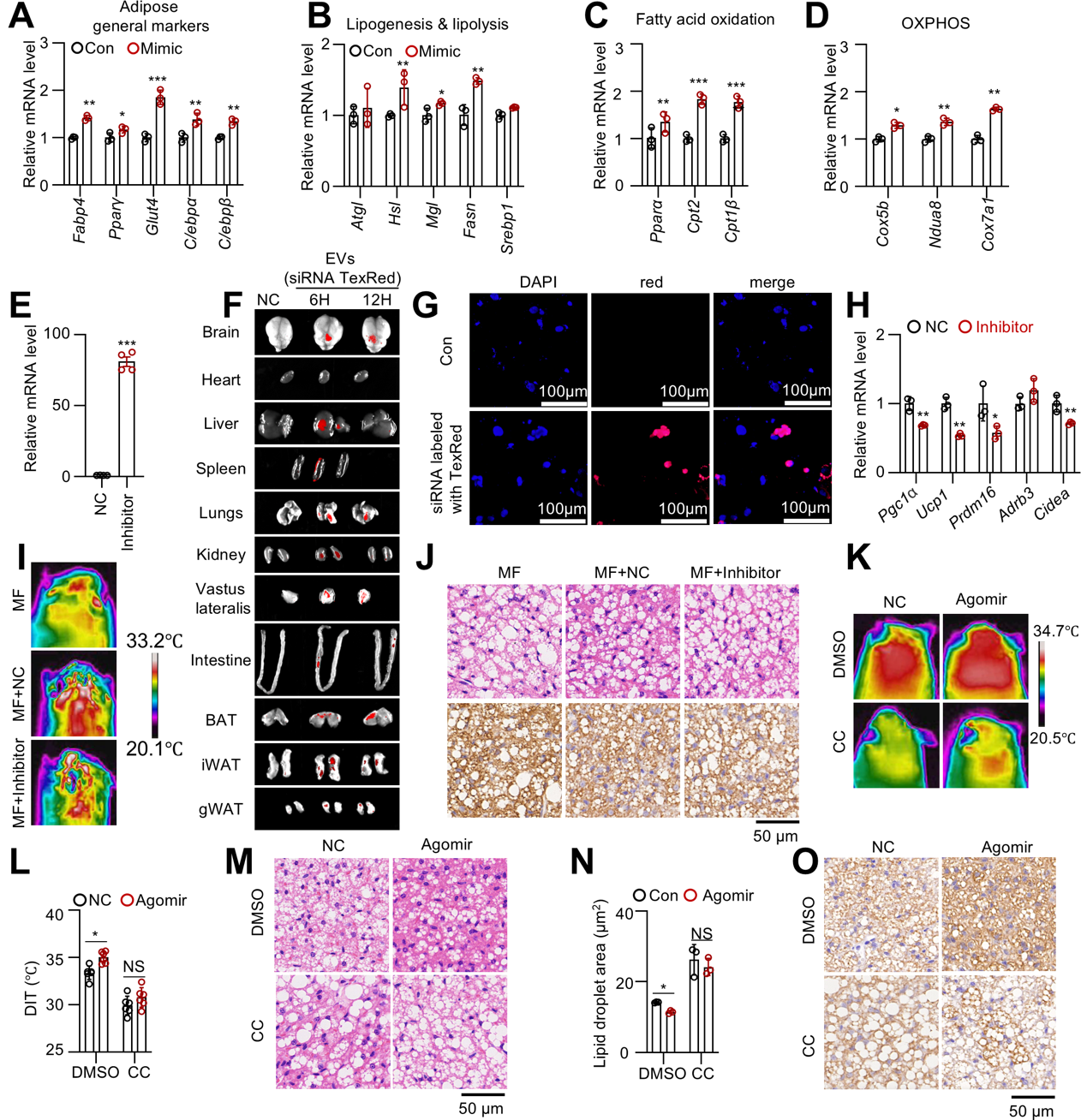


**Supplemental figure 6. miRNA-125a-5p promotes BAT thermogenesis**

A-D. BSVs were transfected with miRNA-125a-5p mimic for 48 hours, and brown adipocyte metabolism-related genes were analyzed using qRT-PCR. (n=3)
E. Milk EVs were encapsulated with miRNA-125a-5p inhibitor, and the encapsulation efficiency was determined by qRT-PCR. (n=4)
F. Milk EVs were loaded with Texas Red–labeled siRNA and orally administered to mice. Fluorescent signals in iBAT were examined at 6 and 12 hours post-gavage.
G. Texas Red-labeled siRNA-encapsulated EVs were added to BSV culture, and after 12 hours, fluorescence was checked.
H. BSVs were transfected with miRNA-125a-5p inhibitor, and the expression of thermogenesis-related genes were examined using qRT-PCR. (n=3)
I, J. MF male mice were orally supplemented with modified EVs as indicated, daily for one week. DIT in response to cold exposure (I), H&E staining, and UCP1 IHC (J) were performed.
K-O. At 2 weeks of age, MF male mice were subcutaneously injected with CC and miR-125a-5p agomir in the interscapular region three times a week for one week. DIT in response to cold exposure (K, L), H&E staining (M, N), and IHC analysis of UCP1 expression (O) in iBAT were performed.

Data are presented as mean ± SEM. Statistical significance was determined using a two-tailed Student’s t-test (*p < 0.05, **p < 0.01, ***p < 0.001).


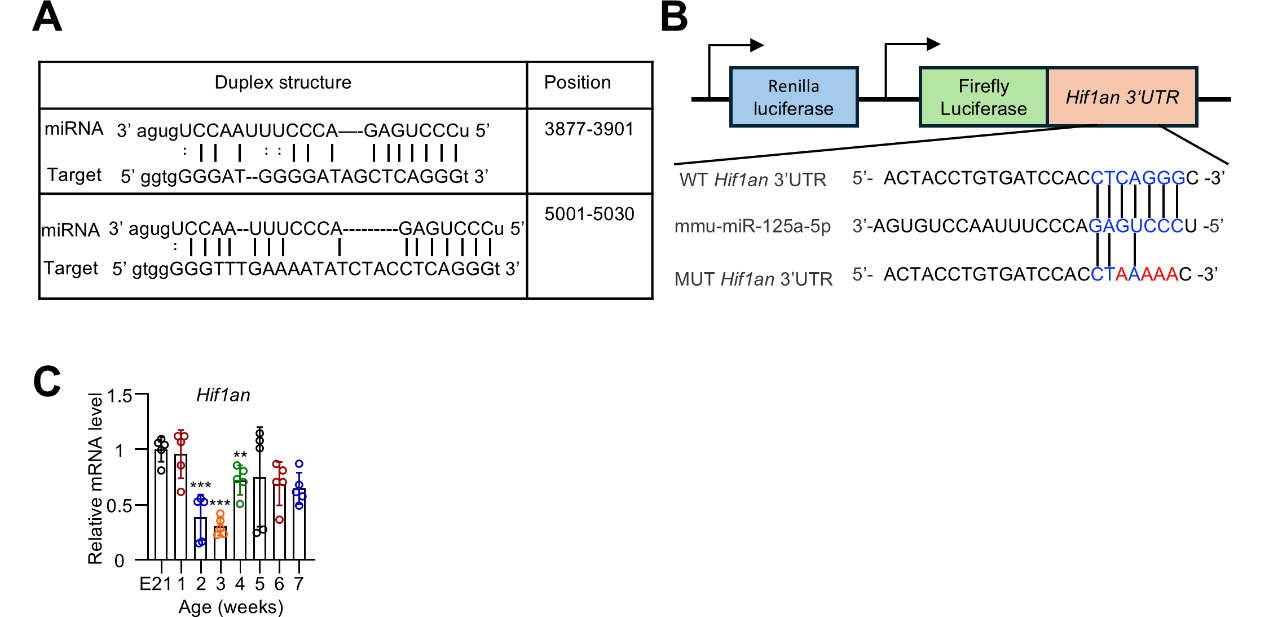


**Supplemental figure 7. miR-125a-5p targets the HIF1AN 3' UTR**

A. HIF1AN contains two highly conserved target sequences that match the 7-mer seed region of miR-125a-5p.

B. Schematic diagram of the HIF1AN 3' UTR firefly dual luciferase construct. The red letters indicate the mutation of four bases to adenine in the HIF1AN 3' UTR sequence.

C. Dynamic changes in *Hif1an* mRNA expression in iBAT at embryonic day 21 (E21) and 7 weeks after birth, as determined by qRT-PCR. (n=5)

Data are presented as mean ± SEM. Statistical significance was determined using a one-way ANOVA (C) (*p < 0.05; **p < 0.01; ***p < 0.001).
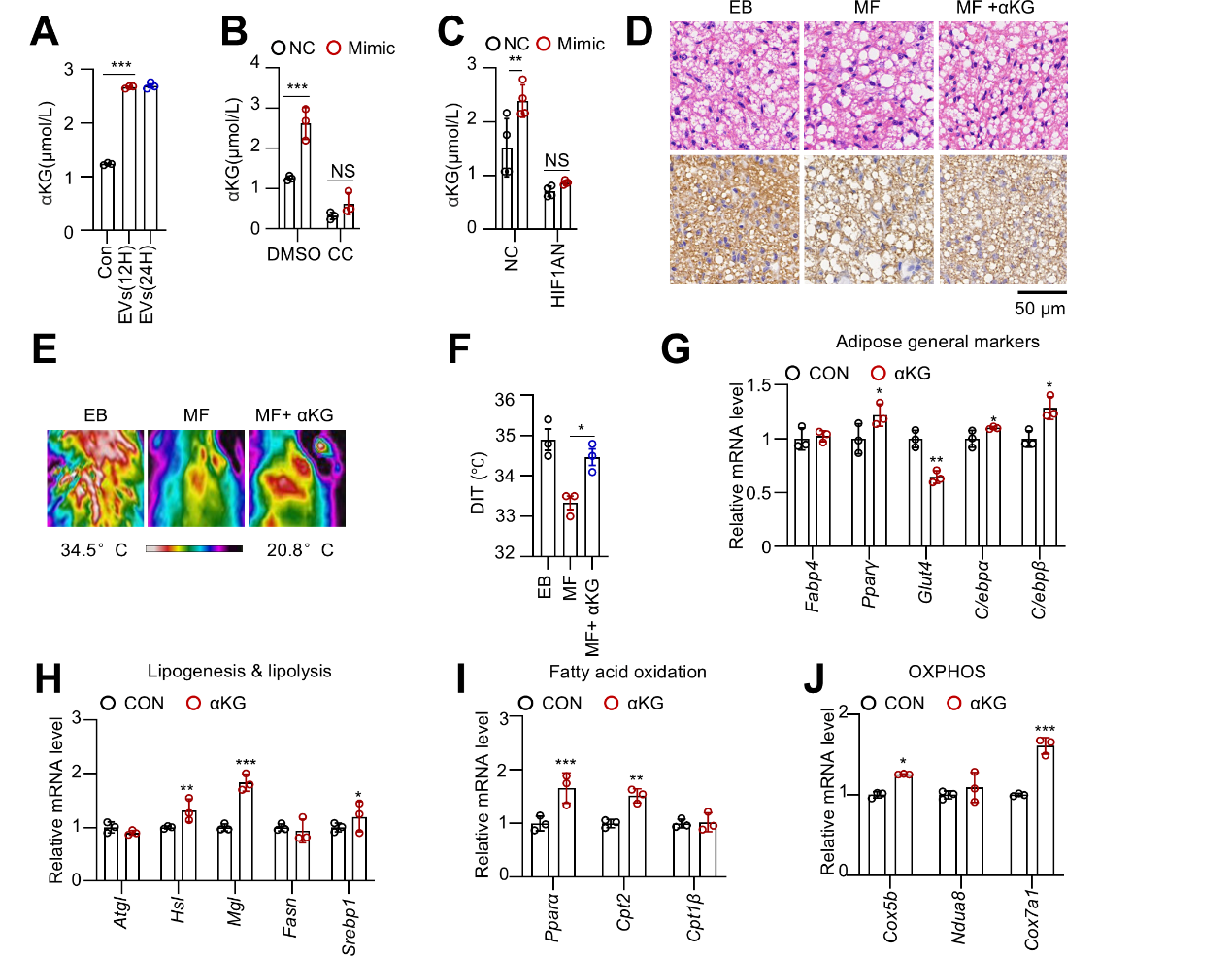


**Supplemental figure 8. αKG promotes brown adipocyte adipogenesis and thermogenesis**

A. BSVs were treated with EVs for the indicated time, and intracellular αKG levels were measured. (n=3)
B. BSVs were transfected with miRNA-125a-5p mimic and treated with CC (20 μM) for 24 hours, followed by measurement of intracellular αKG levels. (n=3)
C. BSVs were transfected with miRNA-125a-5p mimic and a HIF1AN expression construct for 48 hours, and intracellular αKG levels were measured. (n=3)

D. MF mice were injected subcutaneously with αKG, then H&E staining and UCP1 IHC were performed on iBAT tissue.
E, F. MF male mice were subcutaneously injected with αKG (10 mg/kg) in the interscapular region three times per week for one week. DIT was measured in response to 4°C cold exposure. (n=3)
G-J. BSVs were treated with αKG (1 mM) for 12 hours, and the expression of the indicated genes was examined by qRT-PCR. (n=3)

Data are presented as mean ± SEM. Statistical significance was determined using a two-tailed Student’s t-test (*p < 0.05, **p < 0.01, ***p < 0.001).


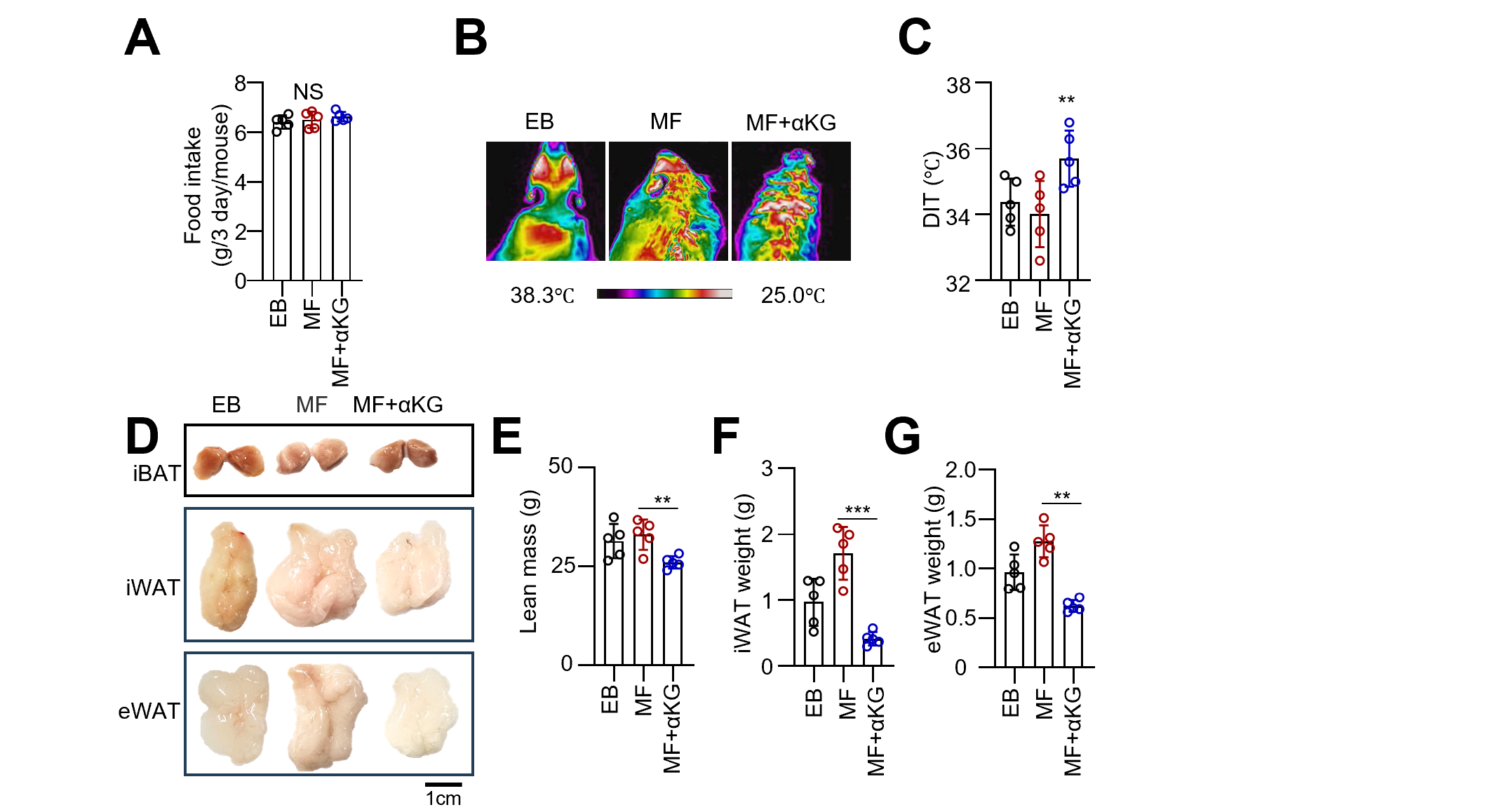


**Supplemental figure 9. αKG restores iBAT thermogenesis in MF mice following weaning**

A. Food intake in EB, MF, and MF+αKG male mice. (n=5)
B, C. After 6 hours of 4°C cold exposure, DIT was measured using infrared thermography. (n=5)
D. After αKG intervention, mice were sacrificed, and iBAT, iWAT, and eWAT were photographed.
E-G. Lean body mass and fat weights in different depots were measured. (n=5)

Data are presented as mean ± SEM. Statistical significance was determined using a two-tailed Student’s t-test (p < 0.05; *p < 0.01; **p < 0.001).

**Supplementary Material.** **The nipples used for artificial rearing**

The nipple used for artificial rearing of 2–3-week-old mice in this study.

Table S1. The Primer sequences used for qPCR.

| Gene name | Forward Primer | Reverse Primer |
| --- | --- | --- |
| *Pparγ* | AACTCCCTCATGGCCATTGA | GCATTGTGAGACATCCCCAC |
| *Glut4* | TCTCCAACTGGACCTGTAAC | TCTGTACTGGGTTTCACCTC |
| *Cebpα* | TACAACAGGCCAGGTTTCCT | AAGTTCCTTCAGCAACAGCG |
| *Pnpla2* | TTGTCCTGCCCCACTAAGAG | CAGGAGTAAGGCACAGGTGA |
| *Lipe* | GTGGTGTGTAACTAGGATTGACTCT | GAACGCTGAGGCTTTGATCTTG |
| *Mgll* | AGTGAGGGAGAGAGGATGGT | AGTCGATGCAGATTCCGGAT |
| *Fasn* | CCGTCGTCTATACCACTGCT | GGCAAAGCTGGTGTCATCAA |
| *Srebf1* | AACGTCACTTCCAGCTAGAC | CCACTAAGGTGCCTACAGAGC |
| *Pparα* | CCACGAAGCCTACCTGAAGA | GGACCTCTGCCTCTTTGTCT |
| *Cpt2* | CAAAAGACTCATCCGCTTTGTTC | CATCACGACTGGGTTTGGGTA |
| *Cpt1β* | CGGTACTTGGATTCTGTGCG | TCTTCCCACCAGTCACTCAC |
| *Ppargc1a* | TCAGAACCATGCAGCAAACC | TTGGTGTGAGGAGGGTCATC |
| *Ucp1* | TGGAAAGGGACGACCCCTAA | CAGGAGTGTGGTGCAAAACC |
| *Prdm16* | CAGCACGGTGAAGCCATTC | GCGTGCATCCGCTTGTG |
| *Adrb3* | CTATGCCAACTCCGCCTTCA | GCCATCAAACCTGTTGAGCG |
| *Cidea* | ATCACAACTGGCCTGGTTACG | TACTACCCGGTGTCCATTTCT |
| *Cebpβ* | GAGCGACGAGTACAAGATGC | GACAGCTGCTCCACCTTCTT |
| *Cox5b* | AGAAGGGACTGGACCCATACA | CCTTTGTGCAGCCAAAACCA |
| *Ndufa8* | GAGTTTATGCTGTGCCGCTG | TACTCTGTGAAAGGCTCCGC |
| *Cox7a1* | TCTTCCAGGCCGACAATGAC | GCCCAGCCCAAGCAGTATAA |
| *Cox6b1* | AACTACCTGGACTTCCACCG | GGTACCACTCACACACGGAG |
| *Gapdh* | CAACTCCCACTCTTCCACCT | GAGTTGGGATAGGGCCTCTC |
| *Fabp4* | AACCTGGAAGCTTGTCTCCA | CACGCCCAGTTTGAAGGAAA |
| *Hif1an* | GTCCCAGCTACGAAGTTACAGC | CAGTGCAGGATACACAAGGTTT |

Table S2. The sequence of the modified 3'UTR of HIF1AN

GGAAAGGTTTCTCACCCTTGCCTCTTGAGCCCCAGAACCTTCCCTCTGCCCCCCTGAAGTCCTGCATTTAGTGTGTGGAGTCCCAGCTTCTGGTTGTAATCATGTCTGTGTTAGTCTGTTAACCTCAGGGTGTGTGTGTGTGTGTACATGTGTGCATATGCATGTGTGTATACACACACATGCATGTATCTGTTCCTTGTTCCCTCTTCCTGGGTCAGGATGTCACTTCTGGCTCTCAGCTCCTGTCTCCTGAAGCCTCAGTGCCTCAGCCTGGGAGGAAGCCTCCCAGTCCTGTGTCTTTCCCTGTCTGAACCACATCCCTGCTCCTGCTGAGCTTCCTGGCTGAGTAGATGAAATGGGGTCAGACTTAGGCAGCTAACTCAATACCTTTCATCCACCTCAGGGCAAGGGGAAAAAAAATGTCCTCTTGCCTCTTAGAGCCAGCGCCTCTGCCAGACCCAACAAAGCGTCGCATGTGAGCTGGAGTAGAGCTGGGAGAGCTTGGAGATAGAATTTACTTTTCTGTTGGTAGCTGTTTATGGGAGCCCTCCTCGGGGCTGCAGCTTATAGCTCTGTGTTATCTCAGTCCTTGGTAAGTTCCCTACCTCTGTTGTCTTTCATCCTTCAAAACTTGGTGCAGAATTTAGCAAGGGCTACCAGCAGTGACCCTCTGACCAGTTTTTCCTACATGGCCAGGGGTGGGGATGGGGATAGCTCAGGGTTGTAGAGGCCAGGCCTTTCCAGTTCCTGTTTCTCTTGGTGTCCTCTTGACCCATCTTTAAGAGGATCCTTGAGCTTTCTCCTAACTAGGCTCTCAGTGGCCTCTTTACCAAGGCTCTTTTCCTCAAAGACGCTCTCTGGCTCCAAGTGCTGGGCAGCAGACAGTCTAGTGTTTAAAAGCATGTGTCTGGGGTCTGGTCCCTTTGGTACATTTCTAGCTTGCTACTTACCAGCTGGGTTTGACCTTGGTTGATTGATTGAACCCGAGTCTCGGTTTCCTTTCCTATAATGACTTGTGGGGGTGGGGGTTTGAAAATATCTACCTCAGGGTTGCTGGATGAACTGAAATAATGTCTGTAAAGCTTTAGCACAGTGCCTGGCAAGCACTTAATAAACGGCTGTGGTGGTGGTGGTTTA

Table S3. Characteristics of study population (n=66).

| Maternal characteristics | Mean±SD |
| --- | --- |
| Maternal age (yeas)  Maternal BMI at delivery (kg/m^2^)  Weight gain during pregnancy (kg)  Birthweight (g)  Gestational age at birth (weeks) | 30.35±4.22  26.83±3.96  11.44±4.58  2715.38±861.96  36.21±3.45 |

Table S4. Composition of the artificial milk

| Ingredient | | Amount(weight/100 ml milk) | Manufacturer | catalog |
| --- | --- | --- | --- | --- |
| Protein(g) | Whey protein isolate (BIPRO) | 4.0 | Davisco Foods | BIPRO |
|  | Whey protein hydrolyzed (PEPTIGEN® IF-3090) | 5.0 | Protein Technologies International | PEPTIGEN® IF-3090 |
|  | Casein (Acid Casein LACTIC CASEIN720) | 4.0 | Fonterra | LACTIC CASE |
|  | Serine | 0.02875 | Sigma-Aldrich | S4250 |
|  | Cystine | 0.3 | Sigma-Aldrich | 285463 |
|  | Tryptophan | 0.027 | Sigma-Aldrich | T9753 |
|  | Methionine | 0.0045 | Sigma-Aldrich | M9375 |
| Carbohydrate (g) | Lactose | 1.89 | Sigma-Aldrich | L5905 |
| Minerals (mg) | NaOH | 25 | Thermo Fisher Scientific | A4782902 |
|  | KOH | 150 | Thermo Fisher Scientific | R21524 |
|  | GlyCaPO_4_ | 800 | Sigma-Aldrich | G6626-100G |
|  | MgCl_2_ 6H_2_O | 190 | Ward's Science | 7791-18-6 |
|  | CaCl_2_ 2H_2_O | 170 | Ward's Science | 10035-04-8 |
|  | CaCo_3_ | 184 | Ward's Science | 1317-65-3 |
|  | Ca-Citrate | 120 | BeanTown Chemical | 5785-44-4 |
|  | Na_2_HPO_4_ | 80 | Sigma-Aldrich | 71643 |
|  | KH_2_PO_4_ | 8 | Sigma-Aldrich | PHR1330 |
|  | FeSO_4_ | 24 | Ward's Science | 7782-63-0 |
|  | Citrate H_2_O | 0.5 | Thermo Fisher Scientific | R21262 |
|  | ZnSO_4_ | 6 | Ward's Science | 7446-19-7 |
|  | CuSO_4_ | 1.5 | Ward's Science | 7758-98-7 |
|  | MnSO_4_ | 0.25 | Ward's Science | 10034-96-5 |
|  | NaF | 0.155 | BDH | BDH9290-500G |
|  | KI | 0.25 | Ward's Science | 7681-11-0 |
|  | K_2_SO_4_ | 163.5 | Ward's Science | 7778-80-5 |
|  | Na_2_SiO_3_ 9H_2_O | 5.075 | Sigma-Aldrich | 307815 |
|  | Na_2_O_4_Se | 0.035 | Sigma-Aldrich | S8295 |
|  | H_8_MoN_2_O_4_ 4H_2_O | 0.0275 | Sigma-Aldrich | 277908 |
|  | KCr(SO_4_)_2_ 12H_2_O | 0.975 | Sigma-Aldrich | 243361 |
|  | LiCl | 0.05 | Sigma-Aldrich | 16398 |
|  | H_3_BO_3_ | 0.285 | Sigma-Aldrich | 15663 |
|  | NiCO_3_ | 0.1125 | BeanTown Chemical | 3333-67-3 |
|  | NH_4_VO_3_ | 0.0225 | BeanTown Chemical | 7803-55-6 |
| Vitamins (mg) | Vitamin mix | 400 | Bio-Serv | 10014-328 |
|  | Vitamin C | 200 | Sigma-Aldrich | 900374 |
|  | Vitamin K3 | 1.9825 | Sigma-Aldrich | 737836 |
|  | Vitamin A | 0.1284 | Sigma-Aldrich | V-010 |
|  | Vitamin D | 23.46 | Sigma-Aldrich | 1717504 |
|  | Vitamin E | 0.0025 | Sigma-Aldrich | 929409 |
| Others (mg) | Carnitine | 4 | Thermo Scientific Chemicals | 6645-46-1 |
|  | Picolinate | 2 | Thermo Scientific Chemicals | 108-89-4 |
|  | Ethanolamine | 3.5 | APOLLO SCIENTIFIC | 77411-750 |
|  | Taurine | 15 | Thermo Fisher Scientific | 101174-114 |
|  | Tricholine citrate | 147 | TCI America | 546-63-4 |
| Fat (g) | MCT | 1.25 | Spectrum Chemicals | 73398-61-5 |
|  | Palm oil | 7.75 | Spectrum Chemicals | 8002-75-3 |
|  | Coconut oil | 2.5 | Thermo Scientific Chemicals | 8001-31-8 |
|  | Soybean oil | 2.75 | Spectrum Chemicals | 8001-22-7 |
|  | Linseed oil | 0.75 | Spectrum Chemicals | 68553-15-1 |
|  | ARASCO® | 0.5 | MedChemExpress | 463-40-1 |
|  | DHASCO® | 0.5 | MedChemExpress | [6217-54-5](https://www.medchemexpress.cn/cas/6217-54-5.html) |
|  | Cholesterol | 0.04 | Sigma-Aldrich | C8868 |
